# Evolving Simple Models of Diverse Intrinsic Dynamics in Hippocampal Neuron Types

**DOI:** 10.3389/fninf.2018.00008

**Published:** 2018-03-13

**Authors:** Siva Venkadesh, Alexander O. Komendantov, Stanislav Listopad, Eric O. Scott, Kenneth De Jong, Jeffrey L. Krichmar, Giorgio A. Ascoli

**Affiliations:** ^1^Center for Neural Informatics, Structures, and Plasticity, Krasnow Institute for Advanced Study, George Mason University, Fairfax, VA, United States; ^2^Cognitive Anteater Robotics Laboratory, Department of Cognitive Sciences, University of California, Irvine, Irvine, CA, United States; ^3^Adaptive Systems Laboratory, Krasnow Institute for Advanced Study, George Mason University, Fairfax, VA, United States

**Keywords:** spiking model, compartmental model, hippocampal neurons, firing patterns, evolutionary algorithms

## Abstract

The diversity of intrinsic dynamics observed in neurons may enhance the computations implemented in the circuit by enriching network-level emergent properties such as synchronization and phase locking. Large-scale spiking network models of entire brain regions offer a platform to test theories of neural computation and cognitive function, providing useful insights on information processing in the nervous system. However, a systematic in-depth investigation requires network simulations to capture the biological intrinsic diversity of individual neurons at a sufficient level of accuracy. The computationally efficient Izhikevich model can reproduce a wide range of neuronal behaviors qualitatively. Previous studies using optimization techniques, however, were less successful in quantitatively matching experimentally recorded voltage traces. In this article, we present an automated pipeline based on evolutionary algorithms to quantitatively reproduce features of various classes of neuronal spike patterns using the Izhikevich model. Employing experimental data from Hippocampome.org, a comprehensive knowledgebase of neuron types in the rodent hippocampus, we demonstrate that our approach reliably fit Izhikevich models to nine distinct classes of experimentally recorded spike patterns, including delayed spiking, spiking with adaptation, stuttering, and bursting. Importantly, by leveraging the parameter-exploration capabilities of evolutionary algorithms, and by representing qualitative spike pattern class definitions in the error landscape, our approach creates several suitable models for each neuron type, exhibiting appropriate feature variabilities among neurons. Moreover, we demonstrate the flexibility of our methodology by creating multi-compartment Izhikevich models for each neuron type in addition to single-point versions. Although the results presented here focus on hippocampal neuron types, the same strategy is broadly applicable to any neural systems.

## Introduction

In the last decade, several projects have built large-scale models of brain regions in an attempt to advance our understanding of how the nervous system functions (Izhikevich and Edelman, 2008; Eliasmith et al., 2012; Markram et al., 2015; Hendrickson et al., 2016). The biological realism in these models has been captured in varying levels of detail. One of the characterizing features of biological neural networks is the diversity observed in the intrinsic dynamics of individual neurons. This diversity likely contributes to the emergent properties of neural networks and, consequently, plays a major role in the information processing in the nervous system (Padmanabhan and Urban, 2010; Tripathy et al., 2013; Pozzorini et al., 2015). Therefore, a biologically realistic large-scale network model of a brain region should take into account intrinsic behavioral diversities both within and between neuron types.

Hippocampome.org is a comprehensive knowledgebase of 122 morphologically identified neuron types in the rodent hippocampal formation (Wheeler et al., 2015). One of the motivations behind developing this knowledgebase was to create a real-scale computational model of the entire hippocampus. Toward achieving this goal, we aim to create individual neuronal models using the electrophysiological and spike pattern properties of neuron types available at Hippocampome.org. In deciding which modeling system to use, we considered simulation costs. High simulation costs of biophysically detailed Hodgkin-Huxley-type neuronal models often impose limits on the scale of network models. Conversely, simpler models, such as leaky integrate-and-fire neurons, cannot capture the wide range of dynamics observed in the hippocampus. Models such as Izhikevich (Izhikevich, 2003) and Adaptive Exponential Integrate-and-Fire (AdEx) (Brette and Gerstner, 2005) have been shown to qualitatively reproduce various firing pattern classes observed experimentally in real neurons, while still being computationally efficient. Therefore, these simpler models with lower simulation costs allow large-scale modeling of biological neural networks in a computationally efficient manner. In this work, we create Izhikevich Models (IMs) that reproduce quantitatively comparable features of various hippocampal spike pattern classes through parameters optimization.

The dynamics of Izhikevich models are highly non-linear and error landscapes that are defined over the resulting parameter spaces typically exhibit properties that make them difficult to optimize, such as multiple local optima. As such, several studies have turned to non-convex, derivative-free optimization methods such as evolutionary algorithms (EAs) to fit a neuronal model's responses to experimentally recorded voltage traces. The models used in these studies range from simple spiking models such as AdEx (Rossant et al., 2010, 2011; Lynch and Houghton, 2015) to biophysically detailed Hodgkin-Huxley type models with multiple compartments (Gerken et al., 2005; Keren et al., 2005; Druckmann et al., 2007; Van Geit et al., 2007). Previous studies have also used various techniques such as a feature-based error function (Druckmann et al., 2007) and a phase plane trajectory density method (Van Geit et al., 2008) to create the error landscape for the EA search. Rössert et al. (2016) created an approach to simplify morphologically detailed microcircuit models to their point-neuron counterparts by applying soma-synaptic correction (to account for dendritic attenuation and delay) and constraining Generalized Integrate-and-Fire neurons around an *in vivo*-like working point. Rounds et al. (2016) used EAs to match firing rates of IMs in a network to experimental recordings in the retrosplenial cortex. However, to our knowledge, optimization techniques have not been successfully used to fit intrinsic IM responses to experimental data. On benchmark optimization tests, the IM showed poor performance compared to other simple models (Rossant et al., 2010, 2011; Lynch and Houghton, 2015). This might be due to the failure to identify an appropriate EA configuration such as the choice of error function and variation operators that are well-suited for the IM parameter space.

Apart from its capability to quantitatively fit IM's responses to experimental voltage traces, the novelty of our automated modeling framework is the integration of spike pattern classification protocols. Previous work (Komendantov et al., in review) identified 23 distinct spike pattern classes overall, among the 89 morphologically distinct hippocampal neuron types in Hippocampome.org for which experimental recordings were available. A behavior for a certain neuron type was defined based on the set of all experimentally recorded spike patterns. If a neuron type exhibited spike patterns of more than one class under different experimental conditions (e.g., bursting and regular spiking for different current stimulation strengths), it was marked as a multi-behavior type. In contrast, a neuron type was marked as a single-behavior type, if all spike patterns recorded from the same neuron under different experimental conditions fell into the same qualitative class. Neuron types with only a single experimentally recorded spike pattern were also marked as single-behavior.

This article presents the modeling approach and results for single-behavior neuron types. We report at least one example for each of the nine distinct single-behavior types, with the goal of illustrating both the approach and the IM's ability to quantitatively reproduce a variety of neuronal behaviors observed in the hippocampus. The single behaviors reported here include spiking with and without frequency adaptation, delayed spiking, bursting, and intermittent spiking or stuttering. In addition to simple point-neuron (single-compartment) models, multi-compartment IMs were created, where the number of compartments varied from two to four depending on the dendritic invasion of a neuron type across hippocampal layers. For example, the somata of hippocampal pyramidal cells in the principal layer extend basal dendrites in the oriens layer and apical trees in the radiatum layer that reach to the lacunosum-moleculare. Thus, these neurons can be represented as 4 compartments, one for each layer (as illustrated in the Methods below). This stratification is important because it segregates the synaptic inputs: distal lacunosum-moleculare dendrites, for example, are the targets of entorhinal projections, while dendrites in radiatum receive intra-hippocampal connections. Although finer morphological variability observed across various neuron types may also contribute to network dynamics, compartmentalized dendritic integration of distinct laminar inputs is likely to play a crucial computational role in cortical circuits. Furthermore, dendrites located in separate layers typically have different active and passive properties from each other and from the soma. A previous large-scale model of the thalamo-cortical system used multi-compartment IMs (Izhikevich and Edelman, 2008). However, that model did not capture the signal transmission properties between the dendrites and soma in a biologically accurate way. In addition, the dendritic compartments did not reflect the appropriate balance of active and passive properties. Another novelty of our automated modeling approach is its capability to create accurate dendritic representations in the multi-compartment IMs. Our dendritic compartments exhibit generally known active and passive properties of the dendrites of real neurons.

## Materials and methods

In this article, a certain spike pattern class will be used to denote a neuron type's “behavior,” since all the neuron types discussed here were examples of single-behavior types. It is worth mentioning that 14 of the 23 distinct spike pattern classes observed in the hippocampus are part of the multi-behavior types and, hence, not reported in this article. Modeling multi-behavior cases requires a different approach, which we are pursuing but remains beyond the scope of this article.

### Spiking model

We reproduced spike patterns by using the nine-parameter variant of the IM (Izhikevich, 2007) because we found that the EA could reliably find better solutions with this IM than the originally proposed four-parameter formalism. IMs have been shown to reproduce qualitatively many spike patterns observed in biological neurons. The state variables membrane voltage (*V*) and membrane recovery variable (*U*) govern this two-dimensional system. The recovery variable *U* approximates the channel kinetics of Hodgkin-Huxley type models (Hodgkin and Huxley, 1952), making it computationally much cheaper to simulate. Parameter “*a”* is the time constant for the recovery variable *U*. Parameter “*b”* defines the degree of coupling between the state variables *V* and *U*. Parameters “*b”* and “*a”* collectively determine whether the model is an integrator or resonator (Izhikevich, 2001). Parameters “*V*_*min*_” and “*d”* are after-spike reset values for *V* and *U*, respectively. Parameter “*k”* defines the shape of the spike upstroke, and *V*_*peak*_ defines the spike cutoff value. Parameters *V*_*r*_ and *V*_*t*_ are resting and threshold voltages, respectively, and *C* is cell capacitance.

(1) $$C\cdot \frac{dV}{dt}=k\cdot (V-V_{r})\cdot (V-V_{t})-U+I$$

(2) $$\begin{array}{l} \frac{dU}{dt}=a\cdot \{b\cdot (V-V_{r})-U\}\\ if\;V=V_{peak}\;then\;V=V_{min},U=U+d \end{array}$$

**Figure 1:**
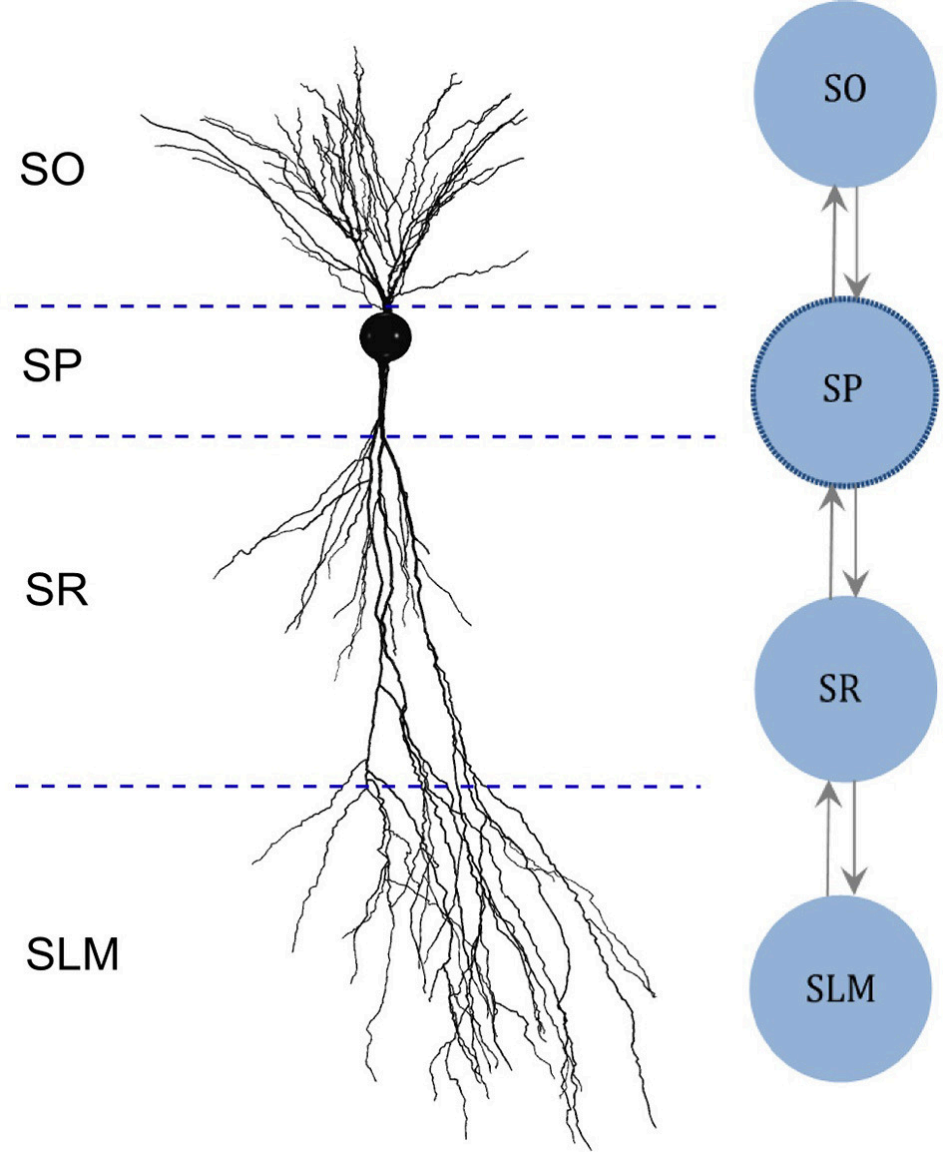
Multi-compartment model layout based on the morphology. **(Left)** Digitally reconstructed morphology of a CA2 pyramidal neuron (Wittner and Miles, 2007), reproduced from Neuromorpho.org (Ascoli et al., 2007). Layer boundaries are approximate. **(Right)** Layout of the multi-compartment model for CA2 Pyramidal neuron type. The number and the layout of compartments are determined based on the invasion of dendrites across the layers of CA2. SO, Stratum Oriens; SP, Stratum Pyramidale; SR, Stratum Radiatum; SLM, Stratum Lacunosum-Moleculare.

In addition, we created multi-compartment (MC) models for each neuron type based on the dendritic invasion across the hippocampal layers (Figure 1), whereas each compartment represents the part of the dendritic tree present in a given layer. Compartments were coupled using an asymmetric mechanism. For example, the MC layout for the CA2 pyramidal neuron type depicted in Figure 1 defines the compartment-specific coupling currents as follows:

(3) $$I_{SP}=G_{1}\cdot P_{1}\cdot \left(V_{SP}-V_{SO}\right)+G_{2}\cdot P_{2}\cdot \left(V_{SP}-V_{SR}\right)$$

(4) $$I_{SO}=G_{1}\cdot (1-P_{1})\cdot \left(V_{SO}-V_{SP}\right)$$

Here, Stratum Pyramidale (*SP*) denotes somatic compartment, and Stratum Oriens (*SO*), Stratum Radiatum (*SR*) and Stratum Lacunosum-Moleculare (*SLM*) (Figure 1) are dendritic compartments. *I*_*SP*_ is the total coupling current at compartment *SP*, which results from the differences in the voltage between *SP* (*V*_*SP*_) and *SO* (*V*_*SO*_), and between *SP* and *SR* (*V*_*SR*_). *G*_1_and *G*_2_ are coupling constants, and *P*_1_ and *P*_2_ (with values between 0.01 and 0.99) determine the degree of asymmetry in the coupling, where a value of 0.5 denotes symmetric coupling.

### Identification of spike pattern classes

To classify the model behaviors, we used the same protocol developed for identifying various transient and steady-state elements of experimentally recorded spike patterns from the hippocampus (Komendantov et al., in review). Transient elements include: Delay (D), if the latency to spike is sufficiently long; Adapting Spiking (ASP), if the spike frequency decreases over time; Transient Stuttering (TSTUT), if a quiescent period follows a cluster of high frequency spikes; and Transient Slow-Wave Bursting (TSWB), if TSTUT is followed by a slow after-hyperpolarizing potential. Steady-state elements include: Silence (SLN), if the quiescence following the last spike is sufficiently long; Non-Adapting Spiking (NASP), if no spike frequency adaptation is identified in non-interrupted firing; Persistent Stuttering (PSTUT), if at least one sufficiently long quiescent period separates two clusters of high frequency spikes; and Persistent Slow-Wave Bursting (PSWB) if slow after-hyperpolarizing potential is present in an otherwise PSTUT pattern.

**Figure 2:**
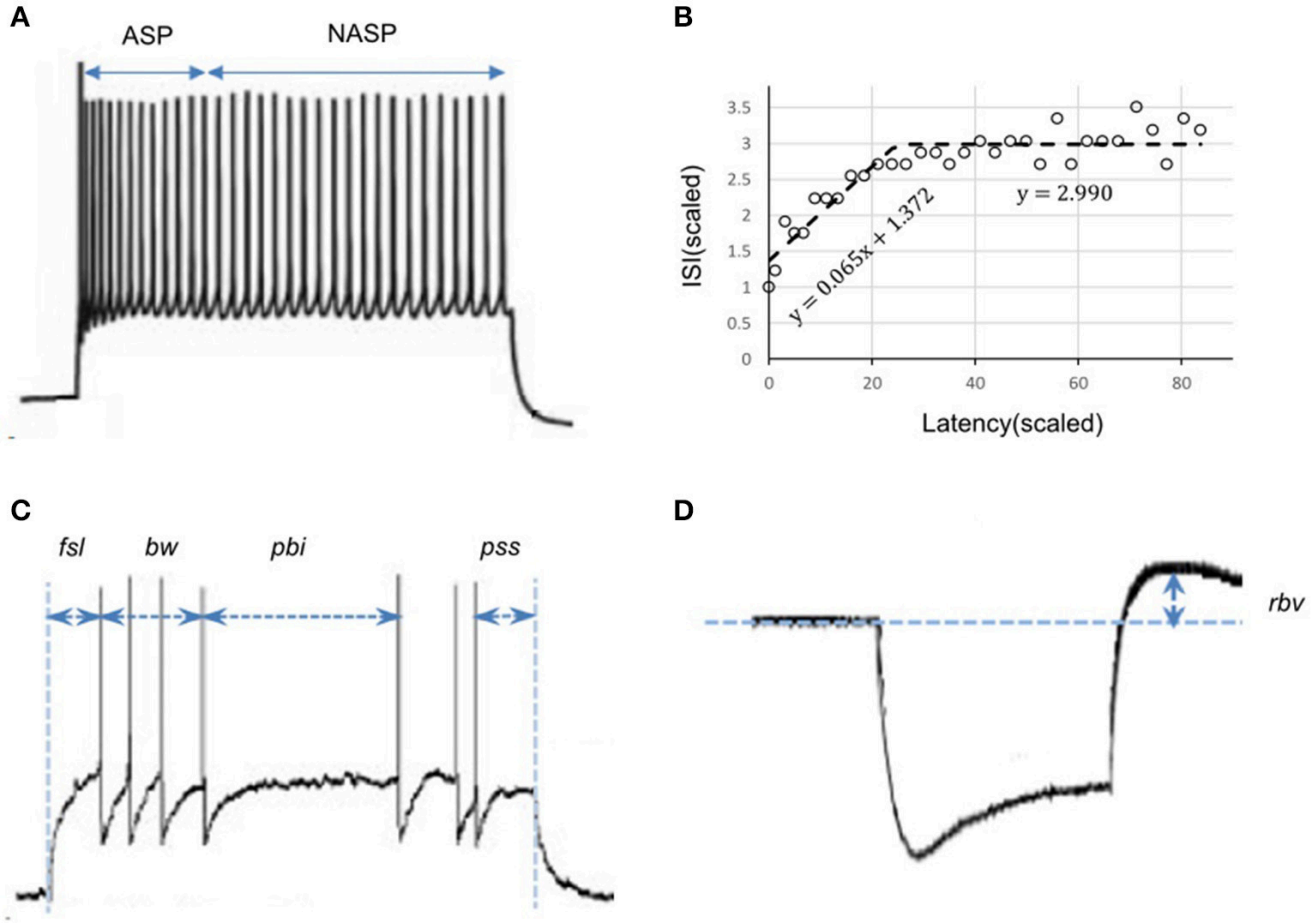
Characterizing features of neuronal spike patterns and subthreshold voltage traces. **(A)** A spike pattern trace recorded from a CA3 Basket-CCK neuron (Gulyás et al., 2010; Hippocampome.org) exhibiting a transiently adapting spiking (ASP.) behavior followed by a steady-state non-adapting spiking behavior (NASP), which is an instance of the class “ASP.NASP.” **(B)** The adapting behavior is quantified by plotting inter-spike intervals (ISI) against their latencies and extracting the parameters of piecewise linear fits, such as slopes and Y-intercepts. **(C)** Stuttering behavior of a CA1 Bistratified neuron (Pawelzik et al., 2002; Hippocampome.org). *fsl*, first-spike latency; *pss*, post-spike silence. The features *bw* (burst width), and *pbi* (post-burst interval) characterizes the stuttering behavior. **(D)** A subthreshold voltage trace recorded from a CA1 OR-LM neuron (Oliva et al., 2000; Hippocampome.org) for a hyperpolarizing current stimulation. The difference between the resting potential and the peak voltage *(rbv)* after the current stimulation stops characterizes the rebound behavior of this neuron.

Given a sufficiently long duration of input current, transients will always be followed by a steady-state in a spike pattern. For example, ASP followed by NASP was identified in the spike pattern experimentally recorded from a CA3 Basket-CCK neuron (Gulyás et al., 2010) and this pattern is an instance of the class ASP.NASP (Figure 2A). The identified class of an experimentally recorded spike pattern represented the target class for model spike pattern. Thus, the criteria that defined a target class were used to validate the model behavior under the given input current. For details on the spike pattern classification criteria, see Komendantov et al. (in review) and Hippocampome.org^1^

### Evolutionary optimization of model parameters

Many varieties of EAs exist along with numerous ways of implementing their specific components (De Jong, 2006). We employed a non-overlapping generational model of evolution and used elitism to ensure that the best individuals were always preserved in the population. In this section, we describe the specific components of the EA.

#### EA configuration

Each individual in the evolutionary population consisted of a complete configuration of the IM we are seeking to tune. We represented these configurations as vectors of floating-points. During the search of the parameter space, we bounded each value within an allowed range. Choosing a biophysically reasonable range for each parameter up front has a significant effect on the efficiency of the optimization procedure, and through preliminary EA runs, we found that different behavior classes required slightly different ranges for some parameters.

When tuning a single-compartment model, each parameter vector contained 9 + *n*_*I*_ elements (genes), representing the 9 parameters of the IM (*k, a, b, d, C, V*_*r*_*, V*_*t*_*, V*_*peak*_*, V*_*min*_) and *n*_*I*_ input currents. *n*_*I*_ equaled the number of voltage traces (which were recorded for different input currents) a model was fit to. A small range encompassing each experimentally injected input current (*I*_*exp*._), [*I*_*exp*._ – *10, I*_*exp*._ + *10] pA* was included in the EA search. By allowing the EA to search within a small range, we boosted the exploration and identified more optimal points (across multiple EA trials) that are very similar in the phenotype. This design also helped to achieve more reliable fitting in cases where a single model was fit to multiple traces (see section Quantitative Comparison of Spike Pattern Features). In rare cases, where the experimental input current was unknown, an unbounded range of [50, 800] pA was included for the search. The multi-compartment models had a larger number of parameters: if *c* is the number of compartments, we require *8c* + 1 parameters representing *c* compartments (the parameter *V*_*r*_ is the same for all *c* compartments), plus *2(c*−*1)* parameters representing coupling parameters for consecutive compartment pairs, and *n*_*I*_ input currents.

We first initialized a population of these vectors by sampling uniformly from within each parameter's allowed range. We used a fixed population size of 120 individuals for single-compartment IMs and of 400 for multi-compartment IMs. An exception was the 4-compartment ASP.SLN fast-spiking model, which we found was easier to optimize with a larger population size (800). After initializing the population, and at each generational cycle thereafter, we immediately selected the 10% of the population with the lowest error to survive to the next generation.

We filled the remainder of the child population by selecting parents via binary tournament and by applying two-point crossover and a mutation operator. Each gene had a probability of mutation between 0.1 and 0.3. For the parameters *d, C, G*, and *I*, we applied an integer random-walk mutation: when selecting one of those genes for mutation, an integer increment or decrement was applied with equal probability. All remaining parameters were mutated by reset: a new floating-point value was randomly chosen out of that gene's allowed range. The EA was run until a maximum number of generations was reached. This number varied between 500 and 5,000 depending on the number of compartments in the model and the class of behavior the model was fit to.

#### Error function

We employed a feature-based error function to quantitatively reproduce the spike pattern. Features for more than 250 experimentally recorded spike patterns are available at Hippocampome.org. These features include first-spike latency (*fsl*), post-spike silence (*pss*), spike frequency adaptation parameters (*sfa*), burst width (*bw*), post-burst interval (*pbi*), and rebound voltage amplitude (*rbv*) (Figure 2). Spike frequency adaptation (*sfa*) was quantified as previously detailed (Komendantov et al., in review) with a piecewise linear regression on the inter-spike intervals (ISIs) by extracting the parameters of linear fits such as slopes (*m*) and Y-intercepts (*c*) (Figure 2B). The error in the model *sfa* was calculated as follows: the two parameters of linear fit (for NASP and ASP. class) or three parameters of piecewise linear fits (for ASP.NASP class) obtained by plotting ISI's against their latencies to the second spike were compared between experimental and model spike patterns. In addition, the number of ISIs (*nisi*) corresponding to a linear fit was also compared. For a bursting/stuttering class, the number of bursts (*nbs*) and the number of spikes (*nspikes*) within each burst were included.

Spike pattern classification protocols were also incorporated into the error function by dynamically assigning different weight factors to different features. This reduced the number of generations required for the EA to find an acceptable solution. In addition, for certain spike pattern classes, this approach more reliably found solutions across multiple stochastic trials. The error function was defined as:

(5) $$error=\sum_{f\in S}\left(W_{f}\times \log\;\left(1+\left|exp_{f}-model_{f}\right|\right)\right)$$

*S*: {*fsl, pss, m, c, nisi*} for continuous spiking, and *S*: {*fsl, pss, nbs, bw, pbi, nspikes*} for interrupted spiking (Figure 2).

Using the spike pattern classification protocols, the qualitative class of a candidate model's spike pattern was first identified. Then, the weight factor *W*_*f*_ was calculated for each feature by comparing the target class with the model spike pattern class. During the EA search, each feature weight changed based on that feature's distance from the target class boundary. These class boundaries are given by the set of criteria that define that class (see Supplementary Material section 1 for pseudocode description of feature weight calculation). This accelerated the search during earlier generations of an EA, when many candidate solutions were outside the target class boundary (fast-exploitation toward narrow regions of interest). Once the population began converging within the target class boundary, this dynamic weight assignment scheme allowed slower exploration and ensured heterogeneity among the best models from within a class (see section Variabilities in the Intrinsic Properties within a Neuron Type).

In order to identify several possibilities, we ran a total of one thousand EA instances for each neuron type, yielding several best models due to the stochastic nature of the EA (different initial populations, stochasticity in variation operators, and selection pressure). At the end of an EA search, the best model was accepted only if its spike pattern class under the given input current matched the target class. If a single-compartment IM failed to reproduce a firing pattern class, two identical compartments were symmetrically coupled. We noticed that coupling effects enriched IM dynamics and were useful to reproduce certain subclasses of stuttering behavior (see Results section for examples).

The parameters of MC models were optimized such that the somatic compartment reproduced features of the experimentally recorded voltage traces both qualitatively and quantitatively using the same techniques discussed previously for single-compartment models. Furthermore, four additional constraints were enforced in order to capture the known general active and passive properties of dendrites in the additional compartments. Unlike the somatic compartment constraints, all MC models shared the same dendritic constraints because of the lack of sufficient experimental dendritic voltage recordings. These general constraints include excitability and input resistance of dendrites relative to the soma as well as forward propagation of spikes and subthreshold signals.

Firstly, the dendritic compartments in MC models were constrained to be less excitable than the somatic compartment when they were decoupled. The minimum depolarizing current (*I*^*rheo*^) required to elicit a spike at a compartment was used as the measure of its excitability (Aou et al., 1992). During the EA search, a ramp current rather than step currents was used to measure compartment excitabilities. This avoided the need for a local search for the minimum step current magnitude required to elicit a spike in each compartment. To reduce capacitive effects in measuring excitability, the ramp current had minimal slopes and high resolution of discrete increments (+0.1 pA/1 ms). Secondly, the decoupled dendritic compartments in MC models were constrained to have higher input resistances than the somatic compartment. The amplitudes of steady state voltage deflections from resting voltage (*V*^*def*^) during a strong hyperpolarizing current input were compared between compartments to measure their relative input resistances. The spike propagation rate (*R*) was defined as the ratio between the number of spikes observed at the destination compartment and the number of spikes initiated at the source compartment. A few hundred excitatory synapses were stimulated at a dendritic compartment for spike initiation. On the other hand, a single AMPA synapse was stimulated at a dendritic compartment and the amplitude of the excitatory post-synaptic potential (*EPSP*) was measured at the somatic compartment. A range of (0.1, 0.9) mV was enforced for the *EPSP* amplitude. All synapses used a value of 10 for the weight, and this value is based on the range used for the multicompartment models by Izhikevich and Edelman (2008). The following errors were calculated for each dendritic compartment and added to the somatic spike pattern error described earlier:

(6) $$error_{rheo}=\{\log\;\left(1+\left(I_{soma}^{rheo}-I_{dend}^{rheo}\right)\right){,}_{I_{dend}^{rheo}<I_{soma}^{rheo}}^{0,\;I_{dend}^{rheo}\geq I_{soma}^{rheo}}$$

(7) $$error_{vdef}=\{\log\left(1+\left(V_{soma}^{def}-V_{dend}^{def}\right)\right){,}_{V_{dend}^{def}<V_{soma}^{def}}^{0,\;V_{dend}^{def}\geq V_{soma}^{def}}$$

(8) $$error_{R}=\{\begin{array}{c} \;0,\;R=1\\ \log\;\left(1+\left(1-R\right)\right),\;R<1 \end{array}$$

(9) $$error_{epsp}=\{\begin{array}{c} \;0,\;0.1\leq EPSP\leq 0.9\;\\ \log\;\left(1+\left(0.1-EPSP\right)\right),\;EPSP<0.1\\ \log\;\left(1+\left(EPSP-0.9\right)\right),\;EPSP>0.9\; \end{array}$$

### Model and algorithm implementations

We used the open-source Java-based evolutionary computation system ECJ (Luke et al., 2015) to tune IM parameters. Single compartment models were simulated using the Apache Commons Mathematics Library^2^. The MC models with up to 39 open parameters were tuned using the parameter tuning interface of CARLsim, an open-source high performance GPU-based spiking neural network simulator (Beyeler et al., 2015). The EA and the single compartment model simulations were run on distributed CPU nodes, and the MC models were run on the GPU nodes available at the Office of Research Computing at George Mason University. All scripts necessary to reproduce the results reported in this article are publicly available^3^.

## Results

### Models of distinct single behavior types

**Figure 3:**
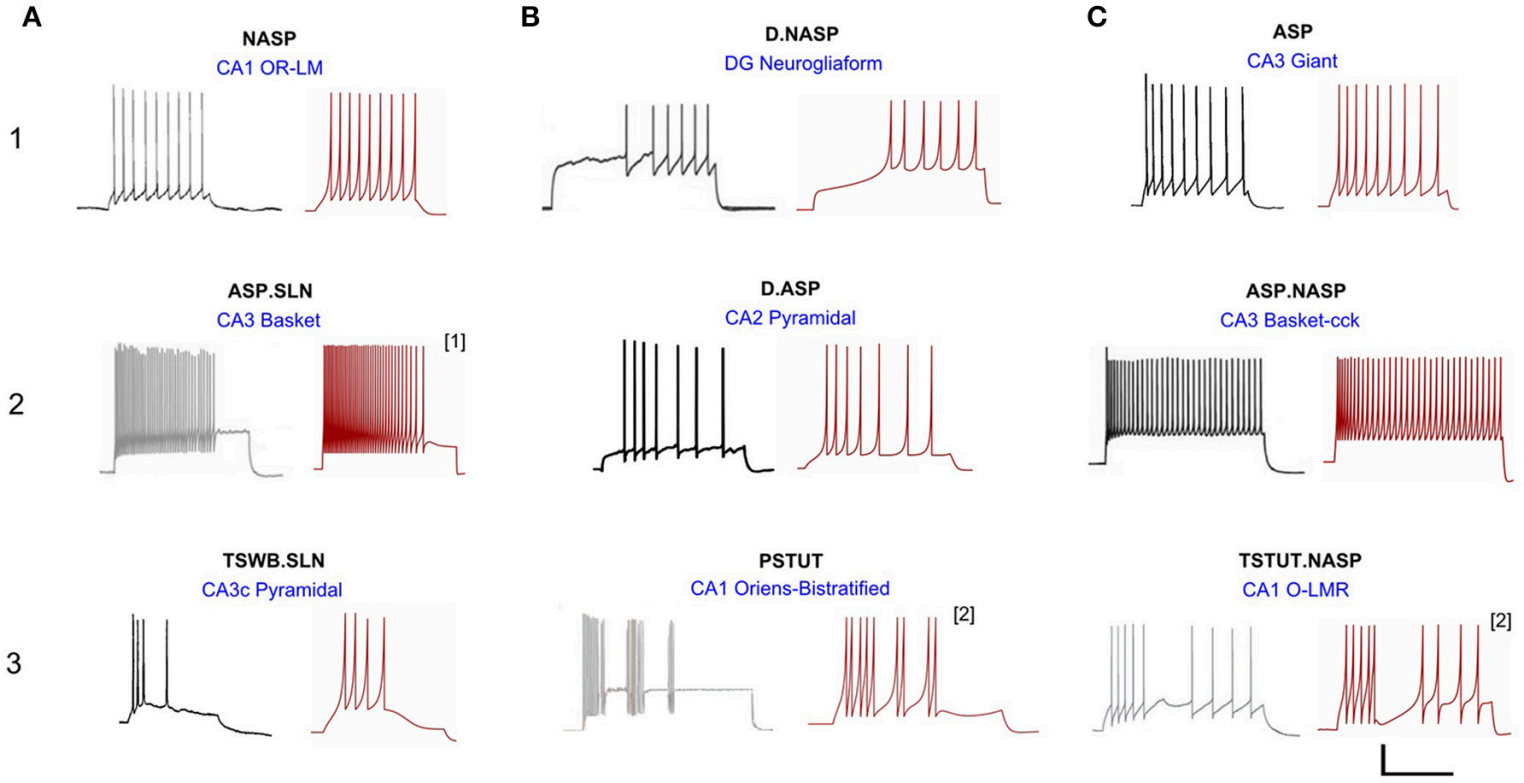
Models reproducing the diverse hippocampal spike pattern classes. Candidate neuron type models for each spike pattern class are displayed as the best IM response across several stochastic EA trials (red traces) along with the corresponding experimental recordings (black traces) digitized by Hippocampome.org from various published sources. The IMs accurately reproduce the features of spike patterns for all classes. Both the experimental and model traces were classified using the same protocols (Komendantov et al., in review). Source of the experimental traces and their calibrations: **(1A)** Oliva et al. (2000); 25 mV, 350 ms. **(1B)** Armstrong et al. (2011); 25 mV, 450 ms. **(1C)** Savić and Sciancalepore (2001); 25 mV, 400 ms. **(2A)** Gulyás et al. (2010); 20 mV, 400 ms. **(2B)** Chevaleyre and Siegelbaum (2010); 20 mV, 200 ms. **(2C)** Gulyás et al. (2010); 25 mV, 300 ms. **(3A)** Buckmaster et al. (1993); 30 mV, 80 ms. **(3B)** Chittajallu et al. (2013); 12 mV, 300 ms. **(3C)** Ali and Thomson (1998); 30 mV, 350 ms. [1] Fast-spiking model with a minimum instantaneous spike frequency of 21 Hz. [2] Two-compartment IM with homogeneous compartments and symmetric coupling. All the other IMs are single-compartment models.

A total of 33 of 122 neuron types in Hippocampome.org version 1.0 (Wheeler et al., 2015) exhibit single behavior. Nine distinct single-behavior *classes* exist among these neuron types, and, in this article, we present at least one model for each of those classes. It is worth mentioning that different neuron types that exhibit the same qualitative behavior class typically exhibit different quantitative features and excitability levels. Figure 3 illustrates an exemplar neuron type for each of the nine distinct single-behavior classes and the corresponding best model from all EA trials. Our simple models were able to reproduce quantitatively comparable spike pattern features for all these classes (see section Quantitative Comparison of Spike Pattern Features). While earlier models reproduced seven qualitatively different classes of spike patterns (Izhikevich, 2003), our models capture the spike pattern features of all observed single-behavior spike patterns in hippocampal neuron types both qualitatively and quantitatively. Importantly, our systematic and more detailed spike pattern classification identifies distinct hippocampal spike pattern classes within general firing behaviors (Komendantov et al., in review). For instance, among the adapting spike patterns, our approach distinguishes between the patterns that reach a specific steady state such as non-adapting or silence (ASP.NASP and ASP.SLN classes, respectively) and those with experimental recordings that only allow determination of the transient state (ASP. class). Our models effectively reproduced the features of these classes (Figure 3: ASP., ASP.SLN, and ASP.NASP).

**Figure 4:**
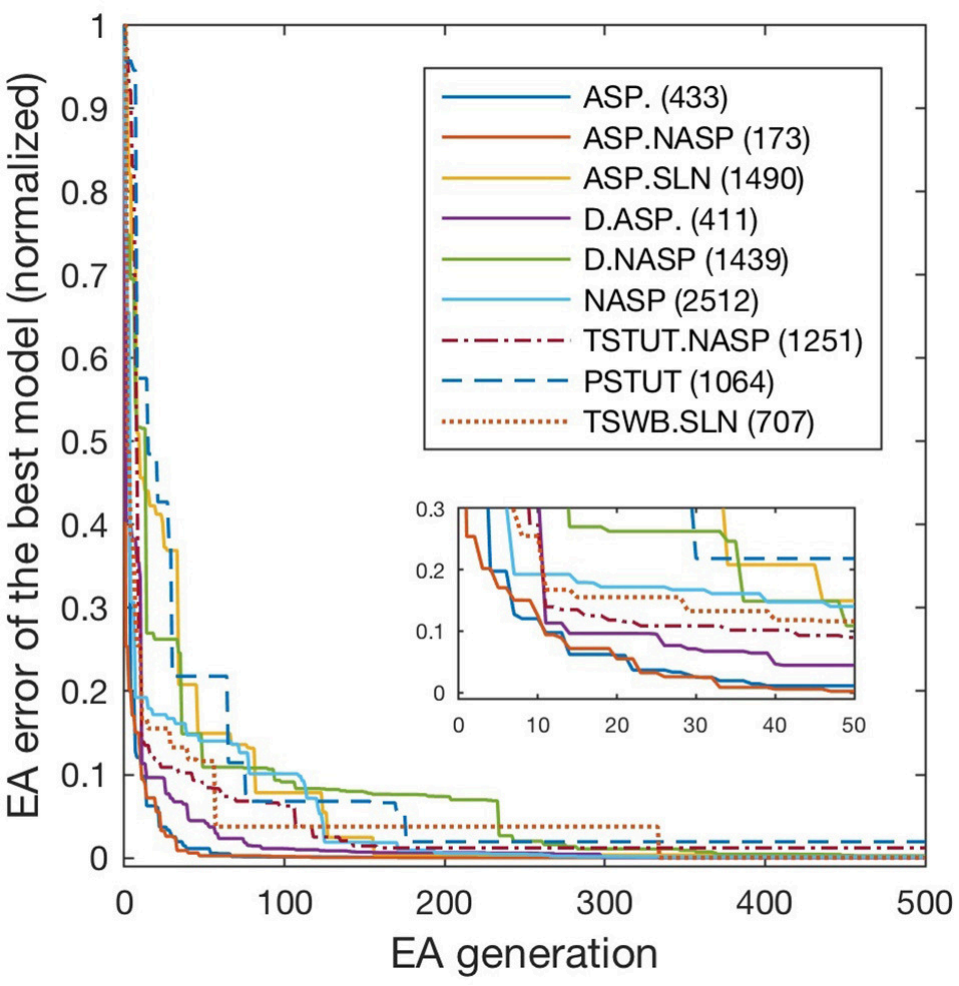
Evolution of best models for different spike pattern classes. EA was run for 500 generations for the classes ASP. and ASP.NASP, 3,000 generations for NASP and ASP.SLN and 2,000 generations for the remaining classes. Errors typically improved at higher rates in earlier generations, when models that satisfy target class criteria were found. Improvements in the error beyond 500 generations were generally small and not shown here. The number next to each class label denotes the last generation of error improvement for that class. Inset zooms-in the first 50 generations.

All models shown in Figure 3 are single compartment IMs except for PSTUT and TSTUT.NASP, which were reproduced by coupling two homogenous compartments. Although stuttering behavior can be modeled in a single compartment IM, multiple compartments (coupling effects) were required to accurately capture various subclasses of stuttering behavior such as TSTUT.NASP and TSTUT.ASP. However, the number of compartments for a multi-compartment IM is determined based on the neuronal morphology (see section Spiking Model) and CA1 O-LMR and CA1 Oriens-Bistratified neurons have both their soma and dendrites in the oriens layer. Thus, MC models were created by symmetrically coupling two identical compartments, unlike the MC IMs with morphologically defined layouts (section Constrained Multi-Compartment Models). These two-compartment IMs were able to capture the classes PSTUT and TSTUT.NASP by integrating coupling effects into the IM dynamics. For the EA search, this simply means inclusion of an additional parameter (coupling constant). In many cases, the EA population converged in less than 500 generations, but certain classes required more generations (Figure 4). As mentioned in section Error Function, we reject the best solution found from a single EA run, if its spike pattern features do not meet the target class criteria (see section Diversity in the Intrinsic Properties across Neuron Types for discussion on the number of accepted models for different classes). The IM parameters of the nine models from Figure 3 are given in Table 1.

**Table 1:** IM parameters for the nine models from Figure 3.

**Neuron type**	***k***	***A***	***b***	***d***	***C***	***Vr***	***Vt***	***Vpeak***	***Vmin***	***G***
CA1 OR-LM (NASP)	0.527	0.00223	6.15	-12	253	−57.25	−42.78	81.81	−44.97	–
DG Neurogliaform (D.NASP)	0.697	0.00107	−30.65	111	242	−74.15	−9.20	17.51	−39.44	–
CA3 Giant (ASP.)	0.609	0.00365	1.84	2	96	−57.58	−37.12	36.42	−49.45	–
CA3 Basket (ASP.SLN)	0.995	0.00385	9.26	-6	45	−57.28	−23.16	18.68	−47.33	–
CA3 Basket-CCK (ASP.NASP)	0.583	0.00574	−1.24	54	135	−59.00	−39.40	18.27	−42.77	–
CA2 Pyramidal (D.ASP.)	5.943	0.00114	−15.89	74	1630	−72.59	−58.78	19.99	−62.65	–
CA1 O-LMR (TSTUT.NASP)	0.326	0.00632	0.40	48	96	−56.44	−27.62	29.48	−51.29	12.00
CA3c Pyramidal (TSWB.SLN)	3.006	0.00189	19.36	104	244	−62.29	−45.27	17.43	−47.37	–
CA1 Oriens-Bistratified (PSTUT)	2.91	0.00168	13.67	35	841	−57.11	−48.50	4.12	−52.94	67.00

### Quantitative comparison of spike pattern features

**Figure 5:**
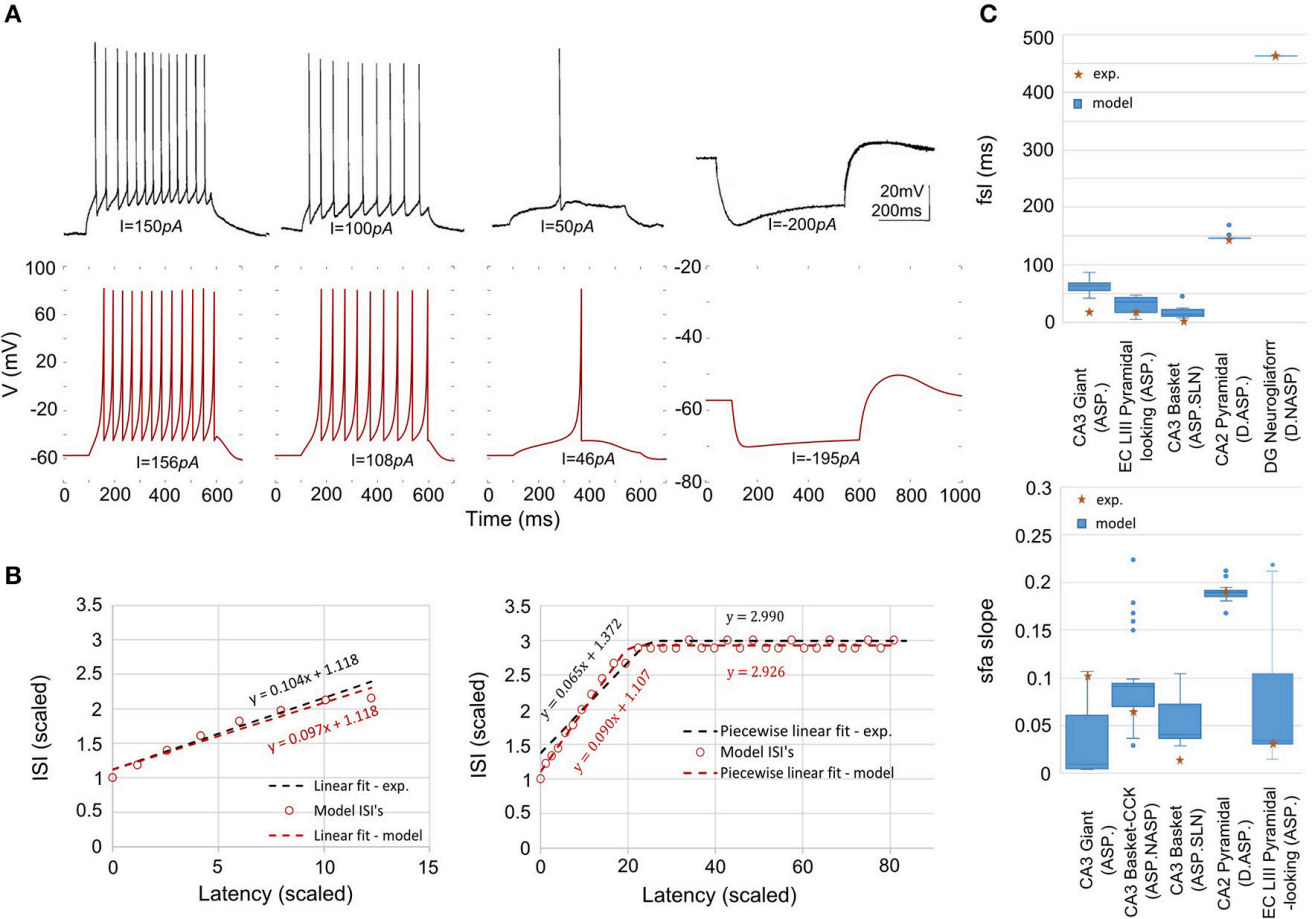
Models quantitatively reproducing the features of experimentally recorded spike pattern traces. **(A)** Experimental recordings from a CA1 OR-LM neuron (Oliva et al., 2000) for four different current stimulation strengths (top). The IM reproduces the features quantitatively for similar input currents (bottom). Refer to Table 2 for numerical comparison. **(B)** The IM *sfa* is fit to the experimentally observed *sfa* from a CA3 Giant neuron to generate a spike pattern of class ASP. (left). The IM quantitatively reproduces the ASP.NASP behavior of a CA3 Basket-CCK neuron (right). The slope(s), Y-intercept(s) and the number of ISI's extracted from the experimental linear fit/piecewise linear fits were used as model constraints. **(C)** The ranges of *fsl*'s (top) and of *sfa* slopes (bottom) exhibited by the accepted IMs for various neuron types.

Our approach can reliably fit a model's responses to multiple experimental voltage traces. As an illustration, the model of a CA1 OR-LM neuron type (a variant of the O-LM interneuron superfamily with dendrites in oriens and axons in both radiatum and lacunosum-moleculare) was created by fitting its responses to four distinct experimental voltage traces recorded for different current stimulation strengths (Figure 5A). The model reproduces features of spike pattern and subthreshold voltage traces that are quantitatively comparable to the experimental traces (Table 2). The model spike pattern features are reported for the input currents that were selected by the EA (see Materials and Methods). In addition, only the minimum set of features required to fully capture the temporal properties of spike patterns were included in the error function. For instance, single spike traces do not require *pss* as an objective feature, when *fsl* and *nspikes* are included. By allowing a narrow range for input current, the EA was able to reliably fit the model responses to multiple voltage traces. Although the voltage sag is not as clearly visible as in the hyperpolarized experimental trace, the corresponding model response nevertheless has a post-inhibitory rebound potential with a 7 mV amplitude. It should be noted that for multiple voltage trace fitting, we only considered traces that were recorded under the same experimental conditions (except for the strength of current stimulation), such as animal species (rat vs. mouse), electrode type (patch vs. sharp), and temperature (room vs. body). As mentioned in section Models of Distinct Single Behavior Types, our approach does not only differentiate between different classes of frequency adapting spike patterns, but also reproduces quantitatively comparable parameters of *sfa* (Figure 5B). See supplementary material (section Materials and Methods) for quantitative comparison between experimental and model traces for all nine classes from Figure 3.

**Table 2:** Quantitative comparison of spike pattern features between experimental and model traces of the CA1 OR-LM neuron type given in Figure 5A.

**Exp**.	***I***		**150 pA**	**100 pA**	**50 pA**	**−200 pA**
	*fsl*		40.1 ms	30.39 ms	200 ms	–
	*pss*		18.38 ms	7.31 ms	–	–
	*sfa:*	*c*	1.176	1.196	–	–
		*nISI*	12	8	–	–
	*rbV*		–	–	–	7 mV
	*nSpikes*		–	–	1	–
**Model**	***I***		**156 pA**	**108 pA**	**46 pA**	−**195 pA**
	*fsl*		58.9 ms	79.9 ms	268 ms	–
	*pss*		8.9 ms	3.1 ms	–	–
	*sfa:*	*c*	1.176	1.198	–	–
		*nISI*	11	8	–	–
	*rbV*		–	–	–	7 mV
	*nSpikes*		–	–	1	–

Furthermore, our approach does not simply identify a single optimal point in the IM parameter space, but instead identifies several possibilities that correspond to the known behaviors of a certain neuron type. The size of such region of possibilities in the parameter space depends on the target behavior class to which the model is constrained as well as the amount of experimental data available for each neuron type. For instance, NASP behavior roughly correspond to the range (0.01, 0.1) for the IM parameter “*a*,” whereas ASP., especially a strongly adapting behavior, significantly reduces the possibilities to the range (0, 0.005) for “*a*.” Similarly, if multiple experimental voltage traces were recorded for different input current strengths, the possibilities in the IM parameter space are reduced.

The variability in the quantitative features among all accepted models is given in Figure 5C. The experimentally observed feature typically lies within the range of features observed in the corresponding models, with few exceptions. For instance, while the IMs for the CA3 Giant neuron type exhibited a range of *sfa* slopes that encompassed the experimentally observed *sfa* slope (Figure 5C bottom), its *fsl* lies outside the model range (Figure 5C top). These models were nonetheless accepted because they all satisfy the criteria for the target class (ASP.).

**Figure 6:**
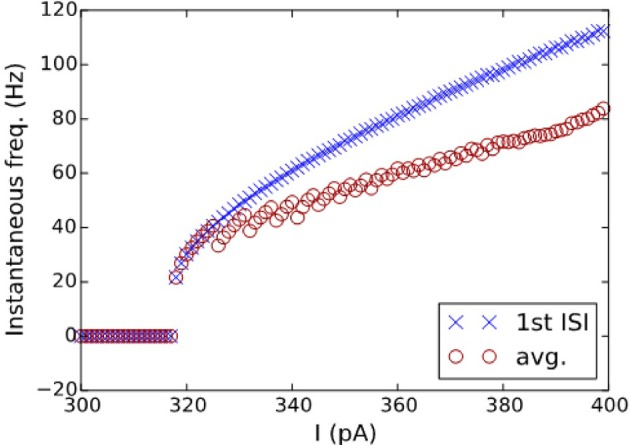
I-F characteristics of the fast-spiking model of a CA3 Basket neuron. Instantaneous frequencies, calculated as the inverse of ISI average (circles) and of the first ISI (crosses), are plotted against the input currents for the model in Figure 3(2A). A minimum frequency of 25 Hz was enforced as a constraint, where the best model found by the EA exhibits a minimum frequency of 21 Hz.

The best model for CA3 Basket (ASP.SLN) showed the highest error in the *sfa* slope among all the adapting classes (Figure 5C bottom). Yet, the accepted models for this neuron type not only exhibited the desired class (Figure 3, ASP.SLN), but also captured fast-spiking behavior, which plays an important role in network synchronization (Traub et al., 1996; Cardin et al., 2009). It should be emphasized that there is no guarantee that a model fit to a single experimentally recorded fast-spiking trace is indeed a fast-spiking model. For example, if the model of a CA3 Basket fast-spiking neuron type was created by simply fitting to the only available trace (Figure 3, ASP.SLN), it might still exhibit non-fast spiking behavior for a lower current input. To avoid this discrepancy, in fast-spiking models we enforced a minimum instantaneous frequency of 25 Hz for a step current close to the rheobase (Figure 6). In the end, a neuron type is represented by a set of heterogeneous models with similar behavioral features.

### Constrained multi-compartment models

In addition to the simple point-neuron models described in previous sections, we create MC Izhikevich models with heterogeneous compartments for all neuron types with dendrites spanning multiple hippocampal layers. These models capture the differences in the active and passive properties between soma and dendrites as well as coupling mechanisms that allow biologically realistic signal transmission between compartments. However, our MC models do not have branching dendritic arbors, and only consists of up to four compartments. This is because each additional compartment adds 10 new parameters for optimization, and tuning hundreds of compartments for each neuron type is an unrealistic goal. We assume that layer-level segregation of synaptic inputs is sufficient to significantly increase the computational power of the models in a network.

**Figure 7:**
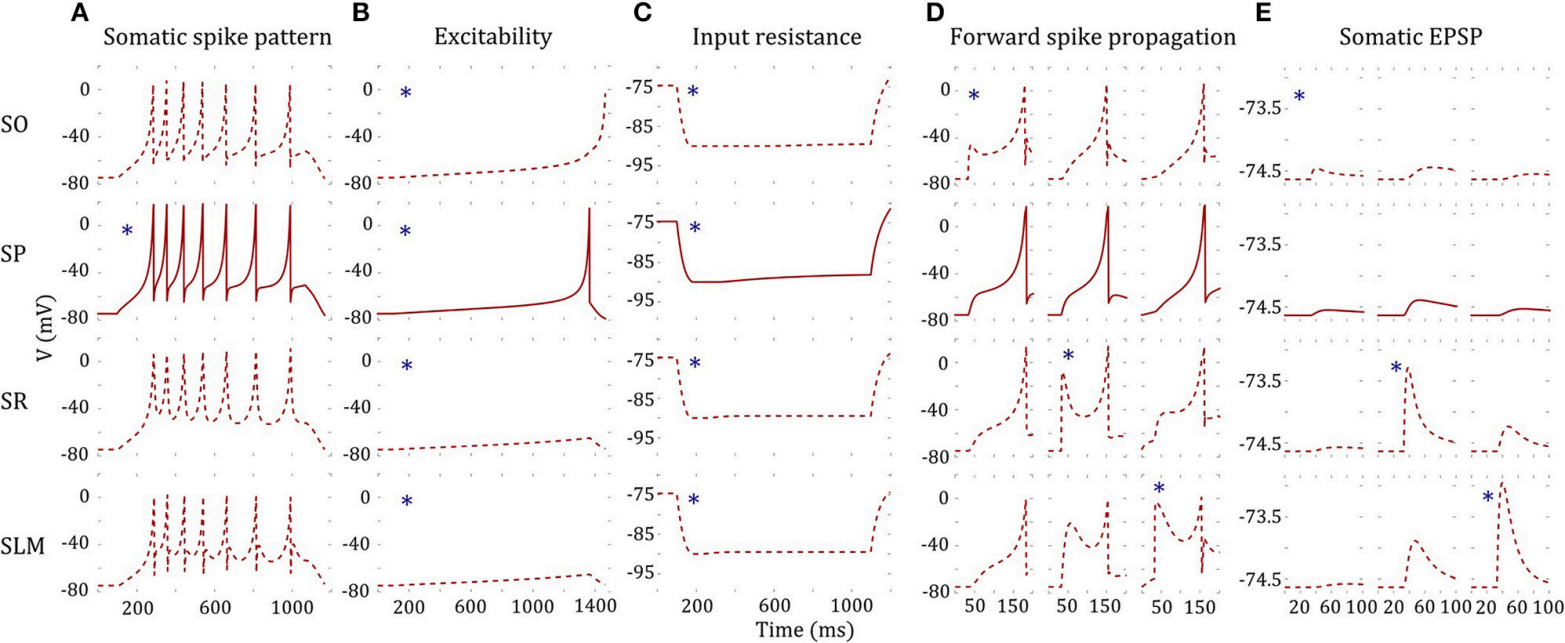
A four-compartment model of a CA2 Pyramidal neuron. ^*^indicates stimulated compartment (using input current/excitatory synapses) **(A)** Somatic compartment reproduces spike pattern of class D.ASP. for I = 401 pA with *fsl:* 188 ms, *sfa*: *y* = *0.173x* + *1.017* [compare with experimental trace in Figure 3(2B) and features in Figure 5C]. **(B)** Decoupled dendritic compartments (DC) are less excitable than the somatic compartment (SC). SC spikes before DC's for ramp current (ramp slope: 0.1 pA/1 ms). **(C)** Decoupled DC's have higher input resistance (IR) than the SC. IR is measured by the steady state voltage deflection due to a hyperpolarizing current application (−500 *pA*). The amplitudes of voltage deflections are 14.92, 13.66, 14.83, and 14.87 mV for SO, SP, SR, and SLM, respectively. **(D)** The model and coupling parameters were optimized to enable forward propagation of spikes to the adjacent compartment. A total of 200 excitatory synapses were stimulated at 40 ms to initiate a spike at a DC. The SLM compartment required an additional input current of 1,200 pA to elicit a spike. Only the forward spike propagation was enforced. **(E)** The model was constrained to evoke a unitary EPSP with amplitude in the range (0.1, 0.9) mV. Excitatory synapses were stimulated at SO, SR, and SLM, and the amplitude of the EPSP was measured at SP. EPSP amplitudes at SP were 0.09, 0.24, and 0.1 mV by stimulating a single synapse at SO, SR, and SLM, respectively.

As an illustration, we present a four compartment model of CA2 pyramidal neuron type (Figure 7). The somatic compartment (SP) reproduced features of experimentally recorded voltage trace [see Figure 3(2B)] both qualitatively and quantitatively (Figure 7A). Furthermore, we enforced four additional constraints for MC models as detailed in section Error Function. Decoupled dendritic compartments are less excitable than the somatic compartment (Figure 7B) and have higher input resistances (Figure 7C). It should be noted that the dendritic input resistances and excitabilities were only enforced qualitatively (the quality of being higher or lower) relative to the somatic compartment. Neither the absolute values nor the magnitudes of the differences were enforced in the error function (see Equations 6 and 7 in section Error Function).

In addition, the dendritic compartments allow forward propagation of spikes to the adjacent compartments. A few hundred excitatory synapses were simultaneously stimulated in order to initiate a spike at a dendritic compartment, and forward spike propagation (in the direction toward soma) was verified at the adjacent compartment (Figure 7D). Interestingly, the SLM compartment required an additional depolarizing current of 1,200 pA in order to initiate a spike, consistent with experimental observations (Jarsky et al., 2005). Although we enforced a spike propagation rate of 1 for isolated spikes initiated at a dendritic compartment (see section Error Function), we noticed that the rate was less than 1 for high frequency dendritic spike trains. Finally, the amplitude of unitary EPSP measured at the somatic compartment was constrained to be in the biologically realistic range of (0.1, 0.9) mV (Figure 7E).

Even though not directly enforced in the error function, our MC models qualitatively exhibited the known directional voltage attenuation properties of hippocampal neurons: voltage attenuation from a dendritic location to the soma is much higher than in the opposite direction (Mainen et al., 1996; Carnevale et al., 1997; Chitwood et al., 1999; Golding et al., 2005). This behavior was observed in the models because of the higher input resistances of the dendritic compartments and the asymmetric coupling between the compartments. The EA always selected weaker coupling toward the soma than away from it. Optimization of a 4-compartment model required ~20 h of total execution time on the GPU. This is roughly a 15X speedup from CPU execution. The 39 parameters of the model from Figure 7 are given in Table 3.

**Table 3:** IM parameters of the 4-compartment model from Figure 7.

**Compartment**	***k***	***A***	***b***	***d***	***C***	***Vr***	***Vt***	***Vpeak***	***Vmin***	***G***	***P***
SO	0.875	0.004	9.154	41	1163	−74.633	−61.327	7.440	−66.761	170	0.407
SP	1.029	0.002	11.054	40	1164	−74.633	−62.009	18.314	−65.184	–	–
SR	0.840	0.016	10.912	42	1174	−74.633	−62.307	14.142	−63.394	169	0.169
SLM	0.833	0.019	9.471	42	1170	−74.633	−60.468	2.444	−66.223	169	0.348

### Variabilities in the intrinsic properties within a neuron type

Our models of hippocampal neuron types were constrained using voltage traces digitized from figures in the published literature. It is thus natural to ask: how faithfully does a representative recording from a single neuron, which the authors chose to include in an article, reveal the real intrinsic property of that neuron type? Under the same experimental conditions, a different neuron of the same type might behave slightly differently due to experimental noise and biological variability. A truly accurate model of a neuron type should take into account (and even represent) such intra-neuron type behavioral variabilities. For instance, if the experimental data consist of spike pattern traces recorded from several neurons of the same type under the same experimental conditions, the error function could use statistical measures such as z-score to capture the variability in the models (Druckmann et al., 2007; Markram et al., 2015). However, we ultimately strive to create spiking models for over a hundred hippocampal neuron types based on the available experimental data as gleaned in Hippocampome.org. Except for a few well-studied neuron types such as CA1 pyramidal neurons, DG granule neurons, and a handful of GABAergic interneurons, the vast majority of neuron types identified in the hippocampal formation to date lack adequate data to represent their behavioral variabilities. While such a paucity of empirical evidence might pose the risk of overfitting the model to experimental noise, two key aspects of our approach synergistically reduce that risk: (i) inclusion of qualitative class criteria in the error function and (ii) parameter space exploration using the EA.

**Figure 8:**
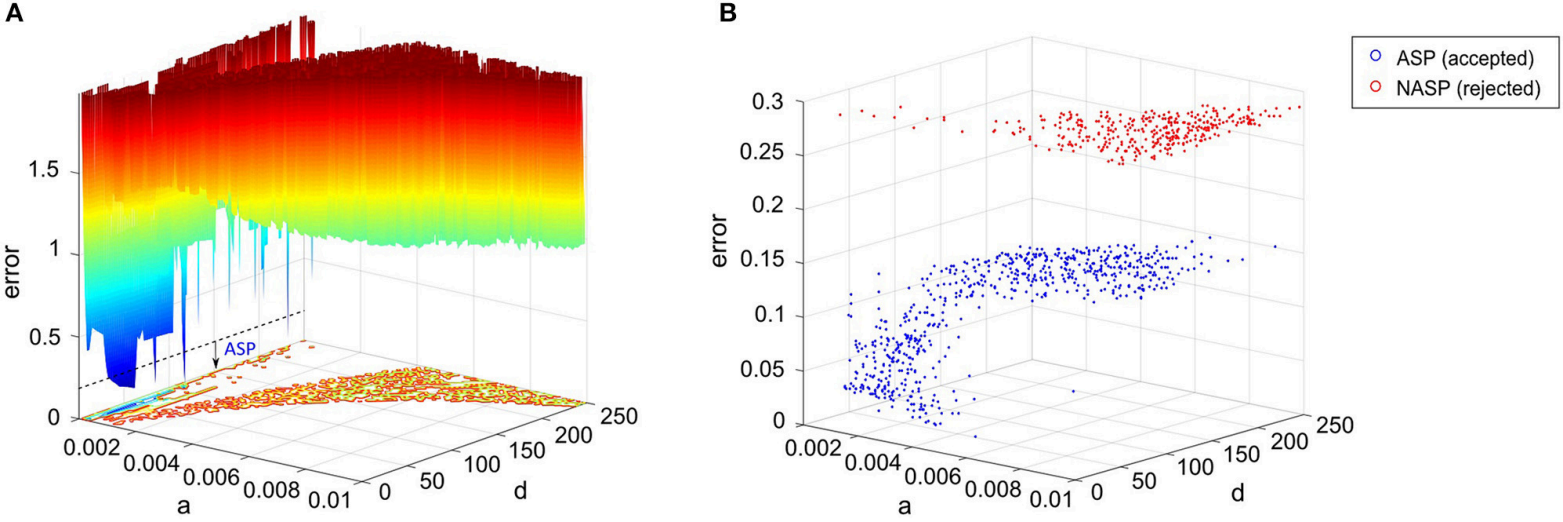
Parameter space exploration by the EA in a landscape that integrates qualitative class definitions. **(A)** Error landscape created by the features of a CA3 Giant neuron that exhibits a spike pattern of class “ASP.” [see Figure 3(1C)]. The dotted line denotes the threshold for model acceptance. All models below this line exhibit quantitative features *similar* to the experimental ones, while strictly adhering to the definitions of the target class (ASP.). This threshold might not necessarily be the same for a different spike pattern of class ASP., since the class definitions were weight factors for the quantitative feature errors rather than separate objectives themselves. The rest of the IM parameters were kept constant to plot this landscape. **(B)** The best models found by the EA across 1,000 stochastic trials for the same neuron type. A total of 651 accepted models satisfied the criteria for ASP., while all rejected models exhibited NASP. These two classes show clear separation in the search space, which is due to the scaling of class-specific feature errors. The EA identified several best models, which are not present in **(A)**, demonstrating exploration capabilities in a multi-dimensional search space. Notice the difference in the “error” axis scale between the two plots.

Firstly, we dynamically weigh the feature errors during the EA search with weights determined by comparing the model's spike pattern class to the experimental target (see section Materials and Methods). This ensures that several near-optimal points in the error landscape represent the appropriate class, even though the exact feature errors might be higher than the globally optimal point (Figure 8A). Without such a weight-assignment scheme, a near-optimal point might not necessarily represent the target class, because both the feature that defines a boundary between classes (e.g., *fsl* between ASP. and D.ASP.) and the feature that does not (e.g., *pss* between ASP. and D.ASP.) would equally contribute to the error. Thus, explicitly integrating qualitative definitions in the error function sharply distinguishes the near-optimal points that satisfy class criteria from the ones that do not (Figure 8B). This increases the EA's chances of finding the models that reproduce the target class.

Secondly, rather than just exploiting the search space to identify a single optimal point that precisely reproduces the spike times, our approach explores the search space and identifies numerous points that elicit a similar behavior. The similarity is governed by the qualitative class definitions, which are inherent to the error landscape as described before. The EA exploration was boosted by a high-rate reset mutation along with a two-point crossover. The downside of such a configuration is the reduced EA reliability in finding acceptable models in certain cases. For instance, only 651 out of 1,000 trials found best models that satisfy the target class criteria (ASP.) for the CA3 Giant neuron type (Figure 8B). It is possible to increase this EA reliability by using a step mutation with a lower rate; however, this will be achieved at the cost of global exploration, ultimately resulting in reduced heterogeneity in the accepted models. In the end, a subset of all best models exhibiting quantitative features with a certain degree of variability is chosen to represent a neuron type (section Quantitative Comparison of Spike Pattern Features). Those features strictly adhere to the criteria for the qualitative class of the spike pattern recorded from that neuron type.

There is no guarantee to avoid over-fitting the best model from a single EA trial to the experimental noise. However, by reducing the acceleration of evolution within the bounds of target class (dynamic feature weight assignment), and identifying several near-optimal points within these bounds (parameter space exploration), we reduce the risk of the best models from all EA trials converging to a single globally optimal point, which might or might not represent a noisy feature. It is worth remembering that a feature threshold of a spike pattern class was statistically inferred from the distribution of that feature from all neuron types (Komendantov et al., in review).

**Figure 9:**
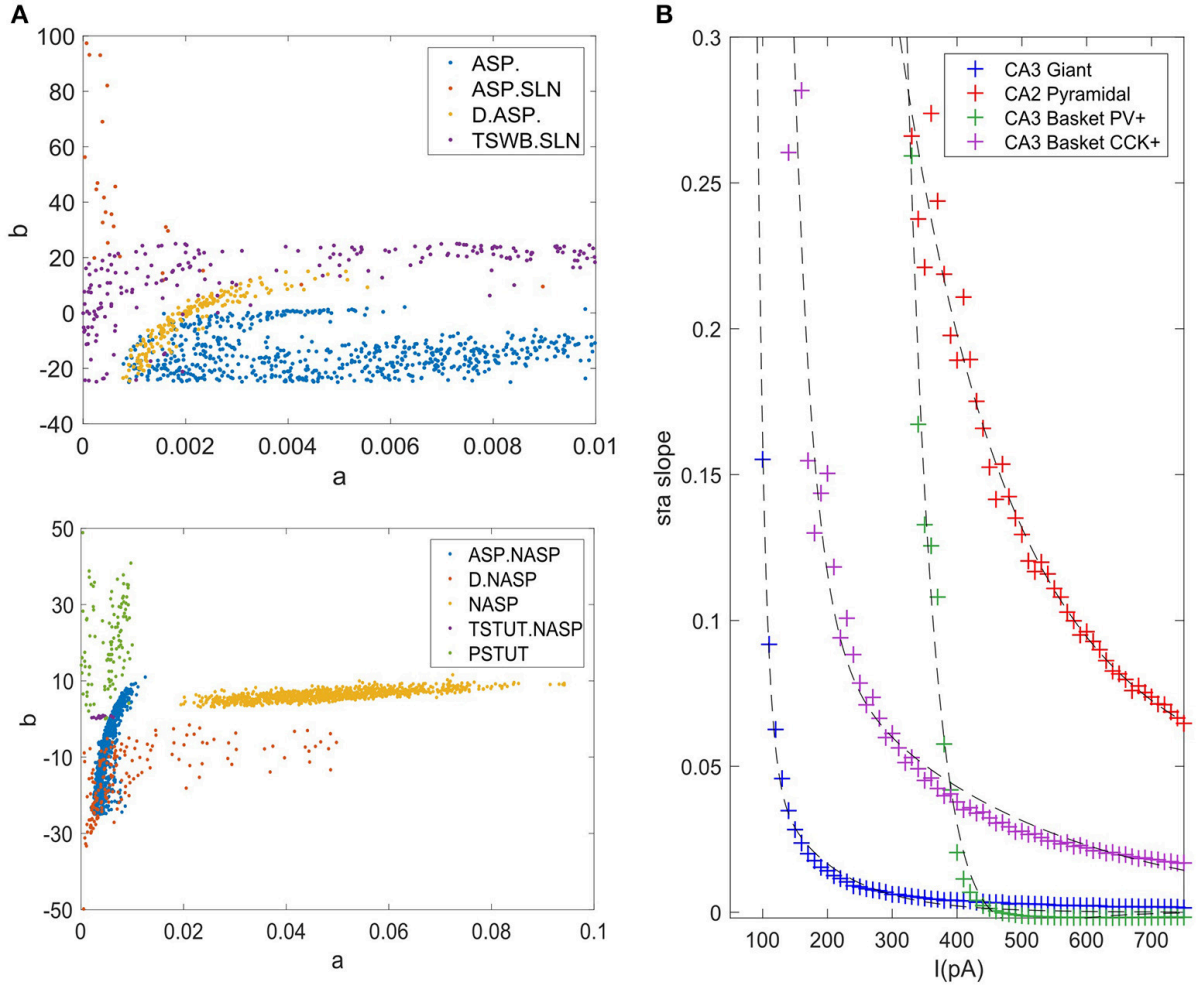
Model and feature variabilities across neuron types. **(A)** Accepted models from each of the nine single behavior types are plotted on dimensions “*a*” and “*b*” of the IM. Best models from all 1000 EA trials were accepted for NASP (bottom), whereas only 25 models were accepted for ASP.SLN with fast spiking constraint (top). All behaviors that include “ASP.” are restricted to the region a < 0.01. Stuttering (PSTUT) and fast spiking (ASP.SLN) behaviors are restricted to the region b > 0 (top). Notice the difference in axes ranges between top and bottom plots. **(B)** The slope of *sfa* is plotted as a function of input current (lasting 500 ms) for the best IMs of four neuron types that included “ASP.” in their behavior. The *sfa* slope decreases exponentially with linearly increasing input step current. These models show substantial variation in their input dependencies of *sfa* slopes.

The accepted models for a single behavior showed notable variation in their parameters, except for TSTUT.NASP (Figure 9A). Such a variation was most prominent for parameters “*a*,” “*b*,” and “*d*,” but only the dimensions “*a*” and “*b*” are shown in Figure 9A for the nine single-behavior types. Thus, a wide range of parameters yielded similar behaviors, demonstrating the robustness of our EA in exploring the parameter space. This is also consistent with the notion that a given neuron behavior may result from multiple distinct combinations of ion-channel conductance densities (Marder and Prinz, 2002).

### Diversity in the intrinsic properties across neuron types

In section Variabilities in the Intrinsic Properties within a Neuron Type, we discussed within-neuron type variabilities, where several IMs for a single neuron type reveal slightly different quantitative features for similar input currents. In addition to this, feature diversities across different neuron types, both qualitative and quantitative, also likely play a major role in the emergent properties in a network.

Even different neuron types that exhibit similar qualitative behavior might reveal substantial diversity in their quantitative features. For instance, the neuron types that include transient ASP. in their behavior are CA3 Giant (ASP.), CA3 Basket CCK (ASP.NASP), CA3 Basket (ASP.SLN), and CA2 Pyramidal (D.ASP.). The magnitudes of *sfa* experimentally observed from these neuron types were ~0.1 for 100 pA, ~0.06 for 400 pA, ~0.02 for 400 pA, and ~0.2 for 400 pA, respectively (Figure 5C). The IMs constrained using these features reveal considerable diversity in the magnitudes of *sfa* among these four models when the input current is gradually increased, as evidenced by plotting *sfa* against a range of input currents “*I*” (Figure 9B). For all cases, *sfa* decreased exponentially with linear increases of “*I*.” However, these models showed notable differences in their excitabilities and their *sfa* ranges. Most of the variance in *sfa* slopes for the fast spiking CA3 Basket PV+ model could be explained by a narrow range of inputs (325–425 pA). This window is much larger for the regular spiking CA2 pyramidal model. Figure 9B illustrates the diversity of input-dependent *sfa* ranges among these four models. Although experimental data are too sparse to validate such ranges of *sfa*s in the models, the above results demonstrate that our approach can create models with remarkable quantitative diversities, even with limited amounts of data.

The diverse single behavior classes were most separated along the dimensions “*a*” and “*b*” of the parameter space (Figure 9A). The four behaviors that include ASP. were restricted to very small values of “*a*” (<0.01), whereas the NASP models converged to a broad range (0.02, 0.1). As mentioned in section Spiking Model, “*a*” is the time constant for recovery variable “*U,*” and lower values for “*a*” results in stronger *sfa*. The region “*b*” > “*a*” correspond to Andronov-Hopf bifurcation (Izhikevich, 2003) and all the fast-spiking ASP.SLN models were identified in the range (9, 90) for “*b*”. Although only 25 best models from 1,000 EA trials satisfied the criteria for ASP.SLN and fast-spiking, these models encompassed a broad range for “*b*” (Figure 9A top).

**Figure 10:**
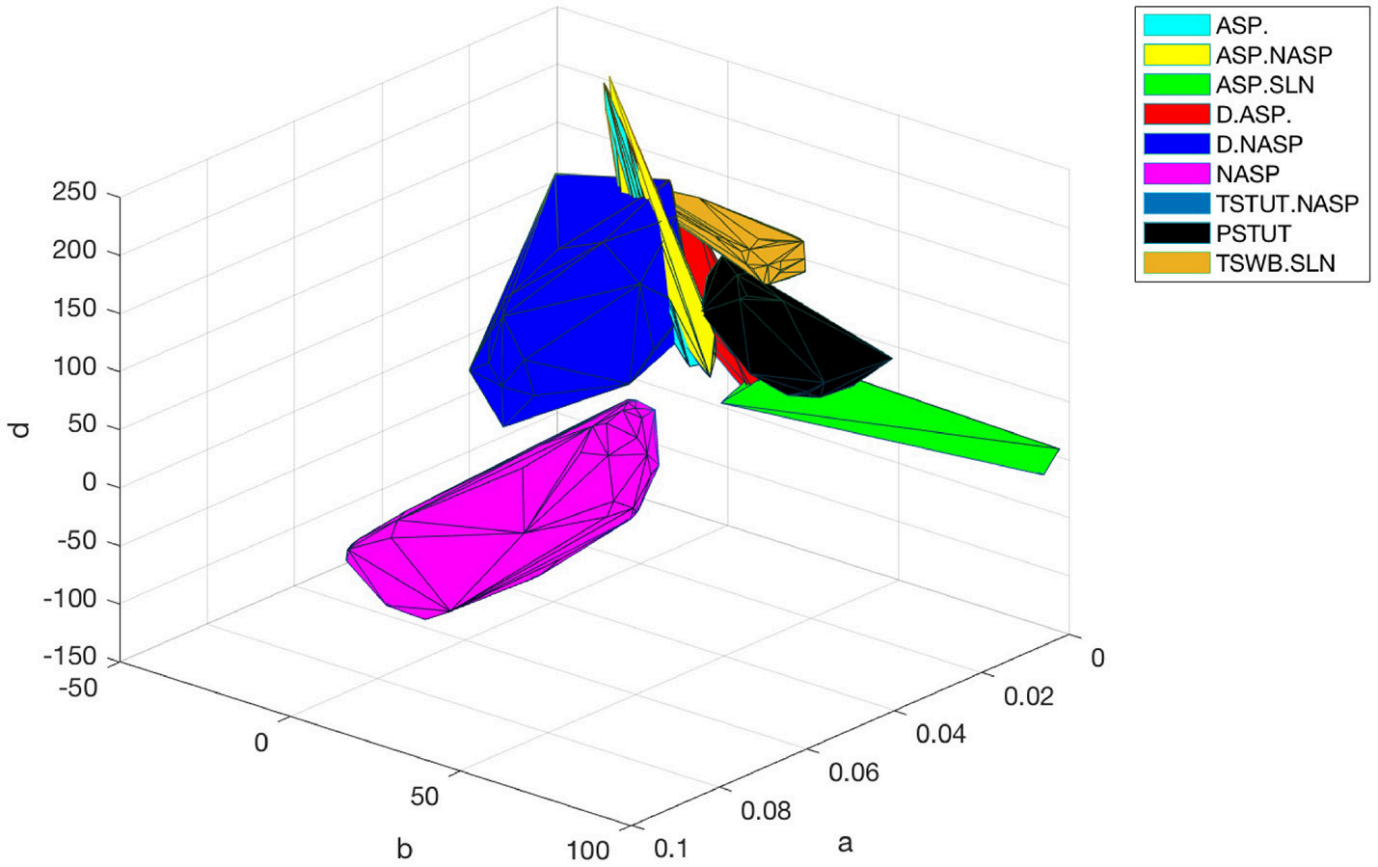
Optimal regions of different spike pattern classes. Region for a class was obtained by plotting the convex hull from all the accepted models. The relationship between parameters “*a*” and “*b*” determines the type of bifurcation and it separates several classes. There is significant overlap between the classes ASP. and ASP.NASP. In general, the EA identified a wide range of optimal points for each class along the dimensions “*a*,” “*b*,” and “*d*”. The classes NASP and D.NASP encompassed larger regions than other classes. The region for TSTUT.NASP, which lies very close to PSTUT (see Figure 9A–bottom) is the smallest region and is not visible here.

The optimal region for each class is shown in Figure 10. There is a significant overlap between the regions for the classes ASP. and ASP.NASP (see also Figure 9A). This is because the difference between these two classes often depends on the input conditions rather than the nine parameters of the model. It is worth mentioning again that in the ASP. class only the transient element is present in the spike pattern. Given a longer duration of input, this pattern will most likely show a steady-state of NASP. The classes NASP and D.NASP encompassed larger regions in the parameter space (Figures 9A, 10). In the case of D.NASP, this is likely due to the fact that the experimentally injected input current was unknown (see Supplementary Table A1), and the EA identified several possibilities for similar behavior under a broad range of input currents.

## Discussion

A major motivation behind the current work is the intent to create large-scale network models using IMs with both biologically realistic within-neuron type behavioral variabilities and experimentally validated between-neuron type diversity. Our compact model representations of diverse neuron behaviors allow the implementation of hippocampal circuit simulations in a computationally efficient manner. More importantly, our results offer a sampling range for a neuron group in a network model (Figures 9A, 10). Several studies have shown that neurons have intrinsic plasticity and undergo homeostatic regulatory mechanisms, which modify their non-synaptic ion-channels such as sodium and delayed-rectifier potassium channels, in order to maintain a certain target activity level in the network (Desai et al., 1999; Aizenman and Linden, 2000; Desai, 2003). This implies similar intrinsic properties or behaviors can arise from various combinations of ion channel conductance densities (Foster and Ungar, 1993; Marder and Goaillard, 2006; Schulz et al., 2006). In the mathematically abstracted IM, this is equivalent to various combinations of parameter interactions. Although it might be difficult to describe such interactions mathematically, a robust EA can identify several optimal points in the multi-dimensional search space that correspond to the known behaviors of a neuron type (Figure 8B). Thus, our method represents a neuron type as possibilities in the model parameter space (Figure 9A). Such a representation is crucial for a thorough and systematic investigation of the contributions of neuronal intrinsic properties to network behavior and function. Our multi-compartment models extend this platform to investigate the effects of dendritic filtering on the emergent network properties, while still being reasonably compact.

Furthermore, the diversity captured in our models may help experimentalists identify and distinguish real neurons in finer electrophysiological terms. Our models of *sfa* suggest the existence of different critical input windows for different neuron types as explained in section Diversity in the Intrinsic Properties Across Neuron Types (Figure 9B). Thus, a neuron exhibiting *sfa* could be characterized by the range of *sfa* slopes and its critical input window. The *sfa* makes a neuron act as high-pass filter (Benda and Herz, 2003) and plays a role in emergent network synchronization (Ermentrout et al., 2001). Two neurons with different input-dependent *sfa* ranges will likely have different filtering properties, and consequently, may contribute to network synchronization in different ways.

An advantage of using a Pareto-optimal front approach for model optimization (Druckmann et al., 2007) is that it avoids the need to weigh different feature errors. However, the performance of such multi-objective optimization techniques is affected by the number of objectives (Khare et al., 2003) and exponentially increasing population sizes are required to represent high-dimensional Pareto-optimal fronts (Deb, 2014). A single interrupted spike pattern trace (e.g., TSTUT.NASP in Figure 3) presents at least eight objectives for optimization (see Supplementary Table A2, CA1 O-LMR). Moreover, some of our simple models are constrained using several spike pattern traces (Figure 5A). With population sizes as small as 120, our approach can efficiently optimize model parameters for several objectives.

On the other hand, the approach created by Rössert et al. (2016) requires a data-driven microcircuit model constructed from morphologically detailed neuron models (such as the one in Markram et al., 2015) as a reference, and such reference models are computationally very expensive. Compared to this approach, our simple models might be less constrained in some cases, but they significantly reduce the open parameter space size to create biologically accurate circuit models. Furthermore, our simplified multi-compartment models intrinsically capture the dendritic voltage attenuation properties without a need for synaptic correction (section Constrained Multi-Compartment Models).

The precise shape of the spike was not captured in some of our models (for example, D.NASP in Figure 3). This could be attributed to the quadratic voltage dependence in the IM voltage equation. The AdEx model (Brette and Gerstner, 2005), which replaces the quadratic term in the IM with an exponential term for the voltage dependence, has been shown to reproduce more realistic spike shapes (Badel et al., 2008). However, our selection criteria for the models were entirely based on the temporal features of the overall spike pattern, and do not include characteristics of individual spikes. More important for the information processing in a neural network are the excitability of neurons, the precise timing of spikes, and the properties of connections. The shape of the spike is unlikely to play an equally prominent role in network dynamics. In fact, in the nine-parameter IM formalism “*k*” and “*Vt*” collectively determine the shape of the spike. It is thus possible to obtain realistic spike shape by restricting the ranges for these parameters (e.g., Figure 3, ASP.SLN). However, we did not explore these parameter ranges and interactions for all the cases for the reasons mentioned above.

Although only IMs have been presented in this article, our framework can be easily enhanced to include any phenomenologically rich model of spiking behavior. The only part of this framework that is specific to the IM is the EA configuration presented in section EA Configuration. This configuration was identified partly based on the topographical features of error landscape created by the IM parameters. Once an appropriate EA configuration is identified, our error function and spike pattern classification procedures are readily applicable to any alternative model.

In the future, we will enhance our framework to model multi-behavior neuron types. At least 15 morphological neuron types in the Hippocampome.org exhibit sharply distinguishable qualitative features under different experimental conditions. One of the commonly occurring multi-behavior types in the hippocampus is stuttering and spiking observed in a single neuron for different current stimulation strengths. For example, a CA1 Bistratified neuron exhibited stuttering and regular spiking behaviors for 400 and 600 pA, respectively (Pawelzik et al., 2002). Similarly, a CA1 Neurogliaform projecting neuron exhibited this multi-behavior for 500 and 700 pA (Price et al., 2005). Our preliminary work with multi-behavior types revealed vast possibilities for modeling such behaviors using IM, which could also provide insights into the existence of electrophysiological subtypes for a given morphological type.

The categorization of neuron type behaviors as either single-behavior or multi-behavior is solely based on the currently available experimental data. Consequently, it is possible that additional qualitative behaviors will be observed in future experiments from neuron type currently considered to display a single-behavior based on available data. An advantage of our modeling approach is that it identifies many possibilities for the known behaviors of a neuron type in the IM parameter space. Furthermore, the flexibility of our framework allows easier addition of newly observed behaviors from a neuron type to improve the accuracy of its model representations. Eventually, we plan to create models for over a hundred hippocampal neuron types and to make them freely available at Hippocampome.org. Nevertheless, although the modeling framework and the results presented in this article pertain to the hippocampus, our approach could be easily adapted to other brain regions.

## Author contributions

SV, AK, and GA created the overall approach and all the models. SL and JK extended the CARLsim framework to include simulation of multicompartment Izhikevich models on the GPU. ES and KD developed the interface between ECJ and CARLsim.

### Conflict of interest statement

The authors declare that the research was conducted in the absence of any commercial or financial relationships that could be construed as a potential conflict of interest.

## Footnotes

^1^http://hippocampome.org/php/Help_Principles_of_Classification_of_Firing_Pattern_Elements.php

^2^http://commons.apache.org/proper/commons-math/

^3^https://github.com/Hippocampome-Org/Time

## References

[B1] Aizenman C. D. Linden D. J. (2000). Rapid, synaptically driven increases in the intrinsic excitability of cerebellar deep nuclear neurons. Nat. Neurosci. 3, 109–111. 10.1038/72049 10649564

[B2] Ali A. B. Thomson A. M. (1998). Facilitating pyramid to horizontal oriens alveus interneurone inputs: dual intracellular recordings in slices of rat hippocampus. J. Physiol. 507, 185–199. 10.1111/j.1469-7793.1998.185bu.x 9490837 PMC2230767

[B3] Aou S. Woody C. D. Birt D. (1992). Increases in excitability of neurons of the motor cortex of cats after rapid acquisition of eye blink conditioning. J. Neurosci. 12, 560–569. 1740692 10.1523/JNEUROSCI.12-02-00560.1992 PMC6575626

[B4] Armstrong C. Szabadics J. Tamás G. Soltesz I. (2011). Neurogliaform cells in the molecular layer of the dentate gyrus as feed-forward γ-aminobutyric acidergic modulators of entorhinal–hippocampal interplay. J. Comp. Neurol. 519, 1476–1491. 10.1002/cne.22577 21452204 PMC4015525

[B5] Ascoli G. A. Donohue D. E. Halavi M. (2007). NeuroMorpho.Org: a central resource for neuronal morphologies. J. Neurosci. 27, 9247–9251. 10.1523/JNEUROSCI.2055-07.2007 17728438 PMC6673130

[B6] Badel L. Lefort S. Brette R. Petersen C. C. Gerstner W. Richardson M. J. (2008). Dynamic IV curves are reliable predictors of naturalistic pyramidal-neuron voltage traces. J. Neurophysiol. 99, 656–666. 10.1152/jn.01107.2007 18057107

[B7] Benda J. Herz A. V. (2003). A universal model for spike-frequency adaptation. Neural Comput. 15, 2523–2564. 10.1162/089976603322385063 14577853

[B8] Beyeler M. Carlson K. D. Chou T. S. Dutt N. Krichmar J. L. (2015). A user-friendly and highly optimized library for the creation of neurobiologically detailed spiking neural networks, in International Joint Conference on Neural Networks (Killarney, IE).

[B9] Brette R. Gerstner W. (2005). Adaptive exponential integrate-and-fire model as an effective description of neuronal activity. J. Neurophysiol. 94, 3637–3642. 10.1152/jn.00686.2005 16014787

[B10] Buckmaster P. S. Strowbridge B. W. Schwartzkroin P. A. (1993). A comparison of rat hippocampal mossy cells and CA3c pyramidal cells. J. Neurophysiol. 70, 1281–1299. 10.1152/jn.1993.70.4.1281 8283200

[B11] Cardin J. A. Carlén M. Meletis K. Knoblich U. Zhang F. Deisseroth K.. (2009). Driving fast-spiking cells induces gamma rhythm and controls sensory responses. Nature 459, 663–667. 10.1038/nature08002 19396156 PMC3655711

[B12] Carnevale N. T. Tsai K. Y. Claiborne B. J. Brown T. H. (1997). Comparative electrotonic analysis of three classes of rat hippocampal neurons. J. Neurophysiol. 78, 703–720. 10.1152/jn.1997.78.2.703 9307106

[B13] Chevaleyre V. Siegelbaum S. A. (2010). Strong CA2 pyramidal neuron synapses define a powerful disynaptic cortico-hippocampal loop. Neuron 66, 560–572. 10.1016/j.neuron.2010.04.013 20510860 PMC2905041

[B14] Chittajallu R. Craig M. T. McFarland A. Yuan X. Gerfen S. Tricoire L. (2013). Dual origins of functionally distinct O-LM interneurons revealed by differential 5-HT3AR expression. Nat. Neurosci. 16, 1598–1607. 10.1038/nn.3538 24097043 PMC3839306

[B15] Chitwood R. A. Hubbard A. Jaffe D. B. (1999). Passive electrotonic properties of rat hippocampal CA3 interneurones. J. Physiol. 515, 743–756. 10.1111/j.1469-7793.1999.743ab.x 10066901 PMC2269181

[B16] Deb K. (2014). Multi-objective optimization, in Search Methodologies, eds Burke E. Kendall G. (Boston, MA: Springer), 403–449.

[B17] De Jong K. A. (2006). Evolutionary Computation: A Unified Approach. Cambridge, MA: MIT Press.

[B18] Desai N. S. (2003). Homeostatic plasticity in the CNS: synaptic and intrinsic forms. J. Physiol. Paris 97, 391–402. 10.1016/j.jphysparis.2004.01.005 15242651

[B19] Desai N. S. Rutherford L. C. Turrigiano G. G. (1999). Plasticity in the intrinsic excitability of cortical pyramidal neurons. Nat. Neurosci. 2, 515–520. 10.1038/9165 10448215

[B20] Druckmann S. Banitt Y. Gidon A. Schürmann F. Markram H. Segev I. (2007). A novel multiple objective optimization framework for constraining conductance-based neuron models by experimental data. Front. Neurosci. 1, 7–18. 10.3389/neuro.01.1.1.001.2007 18982116 PMC2570085

[B21] Eliasmith C. Stewart T. C. Choo X. Bekolay T. DeWolf T. Tang Y.. (2012). A large-scale model of the functioning brain. Science 338, 1202–1205. 10.1126/science.1225266 23197532

[B22] Ermentrout B. Pascal M. Gutkin B. (2001). The effects of spike frequency adaptation and negative feedback on the synchronization of neural oscillators. Neural Comput. 13, 1285–1310. 10.1162/08997660152002861 11387047

[B23] Foster W. R. Ungar L. H. (1993). Significance of conductances in Hodgkin-Huxley models. J. Neurophysiol. 70, 2502–2518. 10.1152/jn.1993.70.6.2502 7509859

[B24] Gerken W. Purvis L. Butera R. (2005). Genetic algorithm for optimization and specification of a neuron model. Conf. Proc. IEEE Eng. Med. Biol. Soc. 4, 4321–4323. 10.1109/IEMBS.2005.1615421 17281191

[B25] Golding N. L. Mickus T. J. Katz Y. Kath W. L. Spruston N. (2005). Factors mediating powerful voltage attenuation along CA1 pyramidal neuron dendrites. J. Physiol. 568(Pt 1), 69–82. 10.1113/jphysiol.2005.086793 16002454 PMC1474764

[B26] Gulyás A. I. Szabó G. G. Ulbert I. Holderith N. Monyer H. Erdélyi F.. (2010). Parvalbumin-containing fast-spiking basket cells generate the field potential oscillations induced by cholinergic receptor activation in the hippocampus. J. Neurosci. 30, 15134–15145. 10.1523/JNEUROSCI.4104-10.2010 21068319 PMC3044880

[B27] Hendrickson P. J. Yu G. J. Song D. Berger T. W. (2016). A million-plus neuron model of the hippocampal dentate gyrus: critical role for topography in determining spatiotemporal network dynamics. IEEE Trans. Biomed. Eng. 63, 199–209. 10.1109/TBME.2015.2445771 26087482 PMC4745257

[B28] Hodgkin A. L. Huxley A. F. (1952). A quantitative description of membrane current and its application to conduction and excitation in nerve. J. Physiol. 117, 500–544. 10.1113/jphysiol.1952.sp004764 12991237 PMC1392413

[B29] Izhikevich E. M. (2001). Resonate-and-fire neurons. Neural Networks 14, 883–894. 10.1016/S0893-6080(01)00078-8 11665779

[B30] Izhikevich E. M. (2003). Simple model of spiking neurons. IEEE Trans. Neural Netw. 14, 1569–1572. 10.1109/TNN.2003.820440 18244602

[B31] Izhikevich E. M. (2007). Dynamical Systems in Neuroscience. Cambridge, MA: MIT Press.

[B32] Izhikevich E. M. Edelman G. M. (2008). Large-scale model of mammalian thalamocortical systems. Proc. Natl. Acad. Sci. U.S.A. 105, 3593–3598. 10.1073/pnas.0712231105 18292226 PMC2265160

[B33] Jarsky T. Roxin A. Kath W. L. Spruston N. (2005). Conditional dendritic spike propagation following distal synaptic activation of hippocampal CA1 pyramidal neurons. Nat. Neurosci. 8, 1667–1676. 10.1038/nn1599 16299501

[B34] Keren N. Peled N. Korngreen A. (2005). Constraining compartmental models using multiple voltage recordings and genetic algorithms. J. Neurophysiol. 94, 3730–3742. 10.1152/jn.00408.2005 16093338

[B35] Khare V. Yao X. Deb K. (2003). Performance scaling of multi-objective evolutionary algorithms, in Evolutionary Multi-Criterion Optimization, eds Fonseca C. M. Fleming P. J. Zitzler E. Thiele L. Deb K. (Berlin, Heidelberg: Lecture Notes in Computer Science; Springer), 2632.

[B36] Luke S. Panait L. Balan G. Paus S. Skolicki Z. Bassett J. (2015). Ecj: A Java-Based Evolutionary Computation Research System. Available online at: https://cs.gmu.edu/~eclab/projects/ecj/

[B37] Lynch E. P. Houghton C. J. (2015). Parameter estimation of neuron models using *in-vitro* and *in vivo* electrophysiological data. Front. Neuroinform. 9:10. 10.3389/fninf.2015.00010 25941485 PMC4403314

[B38] Mainen Z. F. Carnevale N. T. Zador A. M. Claiborne B. J. Brown T. H. (1996). Electrotonic architecture of hippocampal CA1 pyramidal neurons based on three-dimensional reconstructions. J. Neurophysiol. 76, 1904–1923. 10.1152/jn.1996.76.3.1904 8890303

[B39] Marder E. Goaillard J. M. (2006). Variability, compensation and homeostasis in neuron and network function. Nat. Rev. Neurosci. 7, 563–574. 10.1038/nrn1949 16791145

[B40] Marder E. Prinz A. A. (2002). Modeling stability in neuron and network function: the role of activity in homeostasis. Bioessays 24, 1145–1154. 10.1002/bies.10185 12447979

[B41] Markram H. Muller E. Ramaswamy S. Reimann M. W. Abdellah M. Sanchez C. A.. (2015). Reconstruction and simulation of neocortical microcircuitry. Cell 163, 456–492. 10.1016/j.cell.2015.09.029 26451489

[B42] Oliva A. A. Jiang M. Lam T. Smith K. L. Swann J. W. (2000). Novel hippocampal interneuronal subtypes identified using transgenic mice that express green fluorescent protein in GABAergic interneurons. J. Neurosci. 20, 3354–3368. 10777798 10.1523/JNEUROSCI.20-09-03354.2000 PMC6773106

[B43] Padmanabhan K. Urban N. N. (2010). Intrinsic biophysical diversity decorrelates neuronal firing while increasing information content. Nat. Neurosci. 13, 1276–1282. 10.1038/nn.2630 20802489 PMC2975253

[B44] Pawelzik H. Hughes D. I. Thomson A. M. (2002). Physiological and morphological diversity of immunocytochemically defined parvalbumin- and cholecystokinin-positive interneurones in CA1 of the adult rat. J. Comp. Neurol. 443, 346–367. 10.1002/cne.10118 11807843

[B45] Pozzorini C. Mensi S. Hagens O. Naud R. (2015). Automated high-throughput characterization of single neurons by means of simplified spiking models. PLoS Comput. Biol. 11:e1004275. 10.1371/journal.pcbi.1004275 26083597 PMC4470831

[B46] Price C. J. Cauli B. Kovacs E. R. Kulik A. (2005). Neurogliaform neurons form a novel inhibitory network in the hippocampal CA1 area. J. Neurosci. 25, 6775–6786. 10.1523/JNEUROSCI.1135-05.2005 16033887 PMC6725364

[B47] Rossant C. Goodman D. F. Fontaine B. Platkiewicz J. Magnusson A. K. Brette R. (2011). Fitting neuron models to spike trains. Front. Neurosci. 5:9. 10.3389/fnins.2011.00009 21415925 PMC3051271

[B48] Rossant C. Goodman D. Platkiewicz J. Brette R. (2010). Automatic fitting of spiking neuron models to electrophysiological recordings. Front. Neuroinform. 4:2. 10.3389/neuro.11.002.2010 20224819 PMC2835507

[B49] Rössert C. Pozzorini C. Chindemi G. Davison A. P. Eroe C. King J. (2016). Automated point-neuron simplification of data-driven microcircuit models. arXiv:1604.00087v2.

[B50] Rounds E. L. Scott E. O. Alexander A. S. De Jong K. A. Nitz D. A. Krichmar J. L. (2016). An evolutionary framework for replicating neurophysiological data with spiking neural networks, in International Conference on Parallel Problem Solving from Nature – PPSN XIV (Edinburgh: Springer International Publishing), 537–547.

[B51] Savić N. Sciancalepore M. (2001). Electrophysiological characterization of “giant” cells in stratum radiatum of the CA3 hippocampal region. J. Neurophysiol. 85, 1998–2007. 10.1152/jn.2001.85.5.1998 11353016

[B52] Schulz D. J. Goaillard J. M. Marder E. (2006). Variable channel expression in identified single and electrically coupled neurons in different animals. Nat. Neurosci. 9, 356–362. 10.1038/nn1639 16444270

[B53] Traub R. D. Whittington M. A. Stanford I. M. Jefferys J. G. (1996). A mechanism for generation of long-range synchronous fast oscillations in the cortex. Nature 383, 621–624. 10.1038/383621a0 8857537

[B54] Tripathy S. J. Padmanabhan K. Gerkin R. C. Urban N. N. (2013). Intermediate intrinsic diversity enhances neural population coding. Proc. Natl. Acad. Sci. U.S.A. 110, 8248–8253. 10.1073/pnas.1221214110 23630284 PMC3657795

[B55] Van Geit W. De Schutter E. Achard P. (2008). Automated neuron model optimization techniques: a review. Biol. Cybern. 99, 241–251. 10.1007/s00422-008-0257-6 19011918

[B56] Van Geit W. Achard P. De Schutter E. (2007). Neurofitter: a parameter tuning package for a wide range of electrophysiological neuron models. Front. Neuroinform. 1:1. 10.3389/neuro.11.001.2007 18974796 PMC2525995

[B57] Wheeler D. W. White C. M. Rees C. L. Komendantov A. O. Hamilton D. J. Ascoli G. A. (2015). Hippocampome. org: a knowledgebase of neuron types in the rodent hippocampus. Elife 4:e09960. 10.7554/eLife.09960 26402459 PMC4629441

[B58] Wittner L. Miles R. (2007). Factors defining a pacemaker region for synchrony in the hippocampus. J. Physiol. 584, 867–883. 10.1113/jphysiol.2007.138131 17823211 PMC2276992

