# Supplementary material for: Evolving Simple Models of Diverse Intrinsic Dynamics in Hippocampal Neuron Types
Source: Front Neuroinform. 2018 Mar 13;12:8. doi: 10.3389/fninf.2018.00008 (PMC5859109; doi:10.3389/fninf.2018.00008)
Supplement: Supplementary file 1 [file DataSheet1.DOCX]

Supplementary Material

Evolving simple models of diverse intrinsic dynamics in hippocampal neuron types

Siva Venkadesh, Alexander O. Komendantov, Stanislav Listopad, Eric O. Scott, Kenneth De Jong, Jeffrey L. Krichmar, Giorgio A. Ascoli^*^

*** Correspondence:** Corresponding Author: ascoli@gmu.edu

# Feature weight calculation for EA error function

1. Initialize $W_{f}=1,$ for all features
2. Identify model spike pattern class and compare against experimental class
3. IF class mismatch in “D.” ^*^:

$$W_{fsl}= 1+\log\left( 1+\left| DELAY\_FACTOR-\frac{fsl}{\left( {{ISI}_{1}+{ISI}_{2}}/2 \right)} \right| \right)$$

4. IF class mismatch in “.SLN”:

$$W_{pss}= 1+\log\left( 1+\left| SLN\_FACTOR-\frac{pss}{\left( {{ISI}_{n}+{ISI}_{n-1}}/2 \right)} \right| \right)$$

5. IF class mismatch in “STUT/SWB”:

$$W_{pbi}= 1+\log\left( 1+\left| STUT\_FACTOR-\frac{{ISI}_{i}^{max}}{{ISI}_{i+1}} \right| \right)$$

6. IF mismatch in the number of piece wise linear fit parameters ($npw$):

$$W_{m,c,nisi}= 1+\log\left( 1+\left| {npw}_{exp}-{npw}_{model} \right| \right)$$

*Abbreviations:* *ISI* – inter spike interval, *fsl* – first spike latency, *pss* – post spike silence, *pbi* – post-burst interval, *m* – slope, *c* – Y-intercept, *nisi* – number of ISIs.

*Constants:* DELAY_FACTOR = 2, SLN_FACTOR = 2, STUT_FACTOR = 1.5

^*^ IF (experimental class includes “D.” AND model class doesn’t include “D.”)

OR

(experimental class doesn’t include “D.” AND model class includes “D.”)

# Quantitative comparison of spike pattern features for all classes

The relative feature errors and their averages are reported in Table A1 for continuous spike patterns and Table A2 for interrupted patterns from Figure 3. The performance of our EA based approach was at its best for the class D.ASP. with an average relative error of 0.001. The average error was less than 0.8 for all classes except PSTUT. The best model for PSTUT showed an average error of 1.503. It is worth mentioning that the PSTUT model was constrained using a total of eleven features, which include *bw*, *pbi*, and *nspikes* for each burst. In comparison, the class D.ASP. only required five features.

**Table A1.** Quantitative comparison of spike pattern features between experimental and model traces for continuous spike patterns

| Neuron type  (Firing pattern class)  Input current (I) =experiment, model (pA) | fsl *(ms)*  experiment,  model,  *relative error^1^* | pss *(ms)* | m | c | nisis | nspikes | Average error |
| --- | --- | --- | --- | --- | --- | --- | --- |
| CA1 OR-LM^2^  (NASP)  I=150, 156pA | 40.10,  58.90,  *0.469* | 18.38,  8.90,  *0.516* | 0,  0 | 1.176,  1.176,  *0* | 12,  11,  *0.083* | - | 0.214 |
| CA1 OR-LM^3^  (NASP)  I=100, 108pA | 30.39,  79.90,  *1.629* | 7.31,  3.10,  *0.576* | 0,  0 | 1.196,  1.198,  *0.002* | 8,  8,  *0* | - | 0.441 |
| CA1 OR-LM^4^  (NASP)  I=50,46pA | 200.00, 268.00,  *0.340* | - | - | - | - | 1,1,0 | 0.170 |
| DG Neurogliaform  (D.NASP)  I=538pA^5^ | 462.83, 462.83,  *0* | 47.59,  49.33,  *0.037* | 0,  0 | 1.258,  1.258,  *0* | 5,  5,  *0* | - | 0.007 |
| CA3 Giant  (ASP.)  I=100,107pA | 18.62,  44.57,  *1.394* | 41.50,  41.35,  *0.004* | 0.104,  0.097,  *0.067* | 1.118,  1.118,  *0* | 8,  8,  *0* | - | 0.293 |
| CA3 Basket  (ASP.SLN)  I=400, 397pA | 4.29,  10.67,  *1.487* | 200.57,  200.57,  *0* | 0.015,  0.035,  *1.333* | 1.209,  0.705,  *0.417* | 40,  40,  *0* | - | 0.647 |
| CA3 Basket-CCK  (ASP.NASP)  I=400, 408pA | 6.85,  16.00,  *1.336* | 10.10,  7.00,  *0.307* | 0.065,  0.090,  *0.385*;  0,  0 | 1.372,  1.107,  *0.193;*  2.990,  2.926,  *0.021* | 12,  12,  *0;*  20,  20,  *0* | - | 0.280 |
| CA2 Pyramidal  (D.ASP.)  I=400, 407pA | 146.72, 146.73,  *0* | 121.99,  121.99,  *0* | 0.190,  0.189,  *0.005* | 1.045,  1.045,  *0* | 6,  6,  *0* | - | 0.001 |
| CA3c Pyramidal  (TSWB.SLN)  I=500, 510pA | 4.77,  18.00,  *2.774* | 56.56,  48.23,  *0.147* | 0.682,  0.218,  *0.680* | 0.722,  0.992,  *0.374* | 3,  3,  *0* | - | 0.795 |

^1^ $relative error=\frac{\left| experiment-model \right|}{experiment}$. ^2,3,4^ Single model was fit to multiple voltage traces (see Figure 5A and Table 2). ^5^Experimental input current is unknown.

**Table A2.** Quantitative comparison of spike pattern features between experimental and model traces for interrupted spike patterns

| *Features* | CA1 O-LMR^1^  TSTUT.NASP^2^  I=200, 203pA^3^ | CA1 Oriens-Bistratified  PSTUT  I=520, 515pA |
| --- | --- | --- |
| fsl *(ms)* | 43.27, 49.00, *0.132^4^* | 3.88, 56.00, *13.433* |
| pss *(ms)* | 54.03, 43.00, *0.204* | 325.74, 270.08, *0.171* |
| nbs | 2, 2, *0* | 3, 3, *0* |
| bw *(ms):* burst 1 | 161,165,*0.025* | 85,111,*0.303* |
| bw *(ms):* burst 2 | 298,296,*0.007* | 64,26,*0.596* |
| bw *(ms):* burst 3 | - | 23,27,*0.169* |
| pbis *(ms):* burst 1 | 245,245,*0* | 96,96,*0.003* |
| pbis *(ms):* burst 2 | - | 102,102,*0.001* |
| nspikes: burst 1 | 5,5,*0* | 12,5,*0.583* |
| nspikes: burst 2 | 4,4,*0* | 9,2,*0.778* |
| nspikes: burst 3 | - | 4,2,*0.500* |
| Average error | 0.046 | 1.503 |

^1^ Neuron type. ^2^ Spike pattern class. ^3^ (experiment, model). ^4^ (experiment, model, $\frac{\left| experiment-model \right|}{experiment}$).
